# Pirfenidone treatment attenuates fibrosis in autosomal dominant polycystic kidney disease

**DOI:** 10.1172/jci.insight.207552

**Published:** 2026-09-22

**Authors:** Viji Remadevi, Abeda Jamadar, Meekha M. Varghese, Haichun Yang, Sumedha Gunewardena, Darren P. Wallace, Reena Rao

**Affiliations:** 1 Jared Grantham Kidney Institute and; 2 Division of Nephrology, Department of Internal Medicine, University of Kansas Medical Center, Kansas City, Kansas, USA.; 3 Research Service, Kansas City Veterans Affairs Medical Center, Kansas City, Missouri, USA.; 4 Department of Pathology, Microbiology & Immunology, Vanderbilt University Medical Center, Nashville, Tennessee, USA.; 5 Cell Biology and Physiology Department, University of Kansas Medical Center, Kansas City, Kansas, USA.

**Keywords:** Cell biology, Nephrology, Drug therapy, Extracellular matrix, Fibrosis

## Abstract

Autosomal dominant polycystic kidney disease (ADPKD) is a leading genetic cause of kidney failure, characterized by progressive cyst growth, inflammation, and interstitial fibrosis. Renal fibrosis, driven by myofibroblast activation and excessive extracellular matrix (ECM) deposition, is increasingly recognized as a key contributor to disease progression, yet targeted antifibrotic therapies remain limited. Here, we evaluated the therapeutic potential of pirfenidone to suppress fibrosis and disease progression in ADPKD. Single-nucleus RNA sequencing of human ADPKD kidneys identified fibroblasts as the predominant source of fibrous and adhesive ECM, with higher ECM-associated gene expression compared with that in normal kidney fibroblasts. In vitro, primary human ADPKD renal myofibroblasts displayed a similar profibrotic gene expression profile, and pirfenidone treatment suppressed ECM gene expression, cell proliferation, migration, and contractility. In the Pkd1^RC/RC^ mouse model of ADPKD, pirfenidone reduced renal fibrosis, myofibroblast accumulation, ECM deposition, profibrotic gene expression, and associated signaling pathways and improved kidney function. Pirfenidone also reduced kidney enlargement but reduced cyst burden only in female mice. Collectively, these findings demonstrate that pirfenidone attenuates renal fibrosis and improves kidney function in ADPKD by suppressing myofibroblast activation and ECM production, supporting fibrosis as a therapeutic target and highlighting pirfenidone as a potential adjunct to cyst-directed therapies.

## Abstract 2

Beyond targeting cysts, reducing kidney scarring may help preserve kidney function in polycystic kidney disease, highlighting fibrosis as a promising therapeutic target.

## Introduction

Autosomal dominant polycystic kidney disease (ADPKD) is a common inherited monogenic disorder caused by mutations in the *PKD1* or *PKD2* genes. It is estimated that 42.6 per 100,000 persons in the United States and over 12.5 million people worldwide are afflicted with ADPKD (1, 2). Progressive cyst growth in the kidneys and liver, accompanied by interstitial fibrosis, inflammation, and hypertrophy are hallmarks of ADPKD (3). Among these features, kidney fibrosis is a critical pathological component and a major contributor to declining kidney function and eventual progression to end-stage kidney disease (4–7).

Progressive kidney interstitial fibrosis leads to tissue scarring of normal kidney architecture and loss of function and contributes to systemic complications such as hypertension, metabolic dysregulation, and electrolyte imbalance.

In ADPKD, fibrosis is increasingly recognized as an important contributor to disease progression rather than solely a secondary consequence of cyst expansion. ADPKD kidneys show excessive extracellular matrix (ECM) accumulation, abnormal turnover, and extensive remodeling along with activation of profibrotic cell signaling pathways (4–6). This altered fibrotic microenvironment may contribute to cyst growth and disease progression.

Myofibroblasts are the major producers of ECM in chronic kidney disease (8–10). They are highly contractile and migratory cells that express α-smooth muscle actin (αSMA). Myofibroblasts are capable of producing copious amounts of ECM and profibrotic and proinflammatory factors that modify the interstitium in chronic kidney disease and chronic diseases of most solid organs (9, 11). While myofibroblasts are scarce in the normal kidney tubular microenvironment, they are abundant in ADPKD kidneys and often seen surrounding cysts. Our previous studies showed that, in ADPKD kidneys, the cyst-lining epithelial cells drive myofibroblast activation and accumulation via paracrine signaling, establishing a profibrotic pericystic microenvironment (12). Importantly, depletion of myofibroblasts in ADPKD mouse kidneys reduced both interstitial fibrosis and cyst growth, highlighting a unique pathogenic role for these cells in ADPKD (13). ECM accumulation also contributes to cyst growth via integrin signalling (7, 14).

Most patients with ADPKD are diagnosed after cysts have already developed and kidney function has begun to decline. In individuals without a known family history, diagnosis is often triggered by disease-associated complications such as hypertension (13). As a result, many patients have established interstitial fibrosis at the time of diagnosis. Although tolvaptan has become an important disease-modifying therapy for patients at risk for rapid progression, it is typically prescribed for those with more advanced cystic disease (Mayo-Irazabal Imaging Classification 1D and 1E) (14). By this stage, fibrotic remodeling is well established, and ongoing myofibroblast activation and ECM accumulation continue to impair renal architecture and function. Therefore, therapeutic strategies that directly attenuate fibrosis may complement cyst-directed therapies and provide additional benefit in slowing ADPKD progression.

Pirfenidone (PFD; 5-methyl-1-phenyl-2-[1H]-pyridinone) is a pyridine derivative antifibrotic drug approved by the FDA for the treatment of idiopathic pulmonary fibrosis (15). It shows antifibrotic, antiinflammatory, and antioxidant effects across preclinical and clinical models, including fibrotic lesions of the heart, liver, skin, and pancreas (16). PFD is also kidney-protective in animal models of kidney injury, including unilateral ureteral obstruction, nephrotoxicity, diabetic nephropathy, and antiglomerular basement membrane glomerulonephritis, mainly by targeting TGF-β signaling and reducing ECM deposition (17–19).

Given the central role of fibrosis in ADPKD progression, we investigated whether PFD could attenuate kidney fibrosis in the well-established Pkd1^RC/RC^ (RC/RC) mouse model of ADPKD and explored the underlying molecular mechanisms. We found that PFD suppressed myofibroblast activation and ECM deposition, reduced renal fibrosis and kidney enlargement, and improved kidney function. Although its effects on renal cyst growth were less pronounced than its antifibrotic effects, PFD reduced cyst growth in female mice, supporting the concept that targeting fibrosis may provide therapeutic benefit in ADPKD beyond currently available cyst-directed approaches.

## Results

### PFD treatment reduced cell proliferation, migration, and contractility of primary human ADPKD renal myofibroblasts.

**Figure 1:**
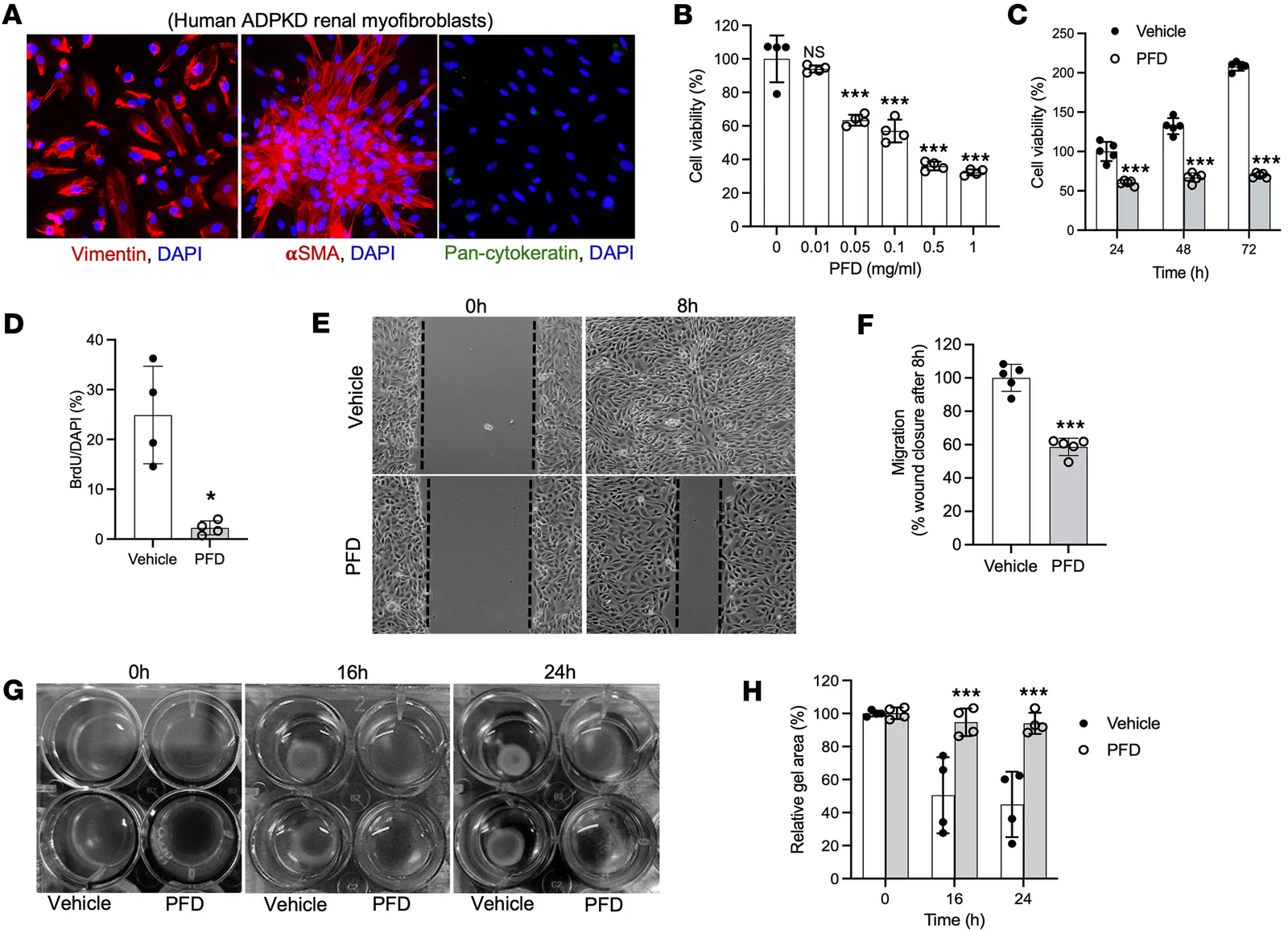
Effect of PFD on primary human ADPKD renal myofibroblasts. (**A**) Human ADPKD myofibroblasts stained for vimentin (denotes mesenchymal cells), αSMA (denotes myofibroblasts), and pan-cytokeratin (epithelial cell marker). (**B**) In human ADPKD myofibroblasts, cell viability assessed by MTT assay after PFD treatment for 24 hours. (**C**) Time course of cell viability in human myofibroblast cells treated with 0.5 mg/mL PFD. (**D**) Cell proliferation measured by BrdU assay after treatment with vehicle or PFD (0.5 mg/mL for 24 hours). (**E**) Migration assay in cells treated with vehicle or PFD (0.5 mg/mL) and (**F**) quantification of migration. (**G**) Gel contractility assay in cells treated with vehicle or PFD (0.5 mg/mL). (**H**) Relative area of the collagen lattice. *P < 0.05, ***P < 0.001 by unpaired *t* test with Welch’s correction. In **B**–**D**, **F**, and **H**, each individual data point represents a biological replicate (primary myofibroblasts) derived from an independent human ADPKD kidney. Original magnification in **A** and **E**: ×20.

To test the effect of PFD in ADPKD, we first tested its effect on primary culture human ADPKD renal myofibroblasts (Figure 1A). PFD treatment reduced ADPKD myofibroblast cell viability (Figure 1, B and C) and cell proliferation indicated by a 10-fold reduction in BrdU incorporation compared with vehicle treatment (Figure 1D). To examine the effect of PFD on migration, a scratch-wound healing assay was performed after mitomycin-pretreatment to block cell proliferation. Vehicle-treated ADPKD myofibroblasts closed the wound within 8 hours, while PFD-treated cells closed only approximately 50% of the wound area over the same time period (Figure 1, E and F). Myofibroblasts express αSMA, contributing to their contractile phenotype and driving tissue distortion and disease progression in solid organs (9). When ADPKD myofibroblasts were cultured in collagen gels, PFD treatment reduced gel contraction by approximately 60% when compared with vehicle treatment at 16 and 24 hours (Figure 1, G and H).

### Fibroblasts are a predominant source of ECM in human ADPKD kidneys.

The cellular sources and composition of ECM in human ADPKD kidneys remain incompletely defined. To identify the cell populations responsible for ECM production, we analyzed publicly available Kidney Interactive Transcriptomics (KIT) single-nucleus RNA sequencing (snRNA-seq) datasets from normal human kidneys (*n* = 5) and ADPKD kidneys (*n* = 8) (20). Genes with an expression value ≥1 were considered actively transcribed.

**Figure 2:**
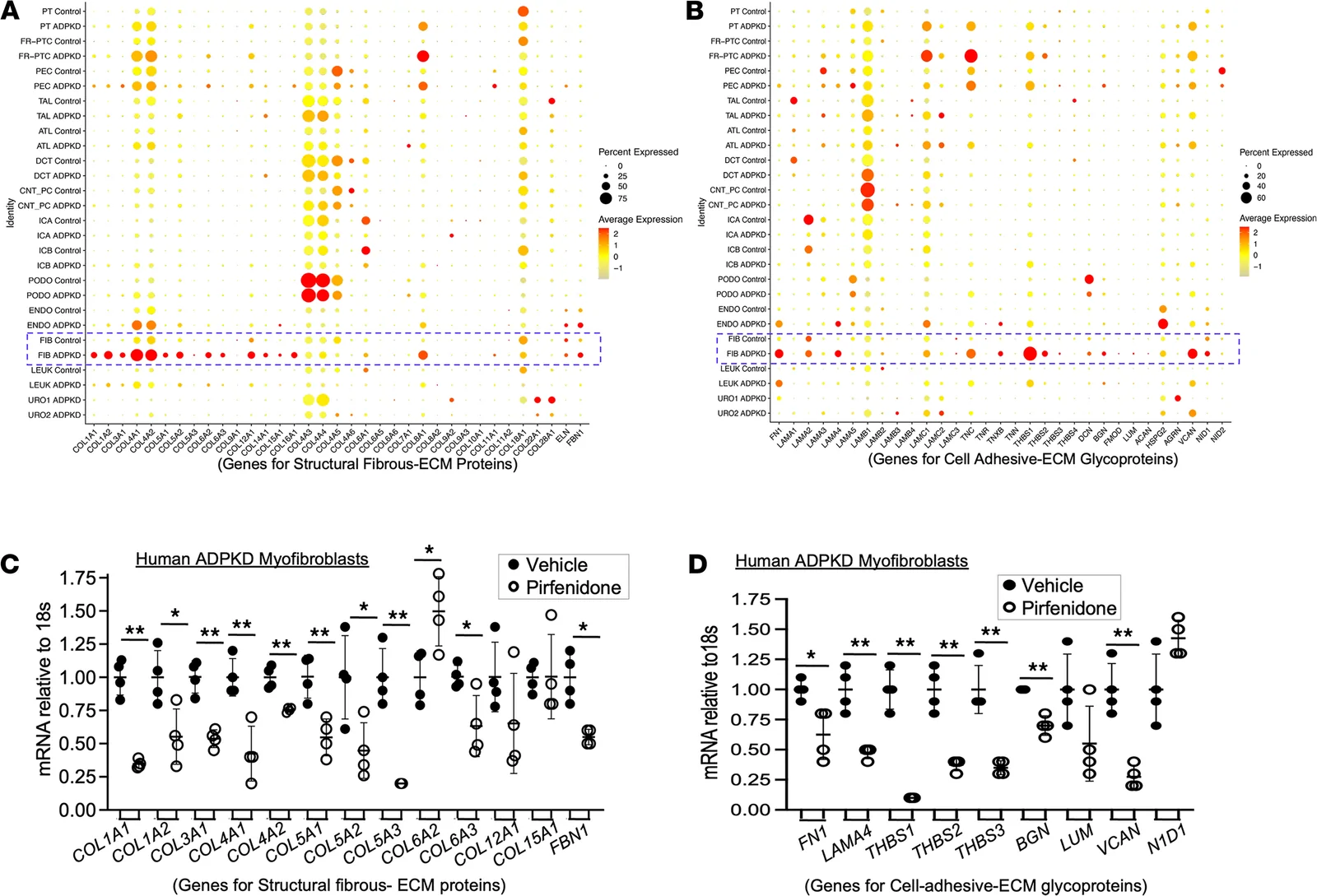
Effect of PFD on expression of structural fibrous-ECM proteins and cell adhesive ECM glycoproteins in human ADPKD renal myofibroblasts. (**A**) Dot plots for human ADPKD (*n* = 8) and human normal control (*n* = 6) kidneys were reanalyzed from KIT snRNA-seq dataset (20). Gene expression for structural fibrous ECM proteins and (**B**) cell adhesive ECM glycoproteins are shown. The size (diameter) of each dot reflects the percentage of cells expressing the gene, while the color intensity indicates the average expression level compared with all cell types. (**C**) Effect of PFD (0.5 mg/mL for 24 hours) or vehicle treatment on mRNA levels of structural fibrous ECM proteins and (**D**) cell adhesive ECM glycoproteins in human ADPKD myofibroblasts. *P < 0.05, **P < 0.01 by unpaired *t* test with Welch’s correction. In **C** and **D**, each individual data point represents a biological replicate (primary myofibroblasts) derived from an independent human ADPKD kidney.

Analysis of structural ECM components revealed expression of 21 collagen subtypes, as well as elastin (*ELN*) and fibrillin1 (*FBN1*), across multiple kidney cell populations (Figure 2A). Among all collagen genes, *COL4A3* and *COL4A4* showed the highest expression and were predominantly localized to podocytes in both normal and ADPKD kidneys. However, fibroblasts emerged as the principal source of fibrillar and interstitial collagens in ADPKD kidneys. Compared with other renal cell populations, the ADPKD fibroblast cluster exhibited the highest expression of 13 collagen genes, including *COL1A1*, *COL1A2*, *COL3A1*, *COL4A1*, *COL4A2*, *COL5A1*, *COL5A2*, *COL6A2*, *COL6A3*, *COL12A1*, *COL14A1*, *COL15A1*, and *COL16A1* (Figure 2A). In addition, several fibroblast-enriched ECM genes, including the above-mentioned collagen subtypes as well as *COL8A1* and *FBN1*, were expressed at higher levels in ADPKD fibroblasts compared with normal kidney fibroblasts (Figure 2A).

We next examined the expression of adhesive ECM glycoproteins. Of the 18 glycoprotein genes detected in ADPKD kidneys, fibroblasts were the predominant source of several key matrix components, including fibronectin (*FN1*), laminin α4 (*LAMA4*), tenascinXB (*TNXB*), thrombospondin1 (*THBS1*), thrombospondin2 (*THBS2*), biglycan (*BGN*), versican (*VCAN*), and nidogen1 (*NID1*) (Figure 2B). Furthermore, expression of multiple adhesive ECM genes, including *FN1*, *TNC*, *LAMC1*, *LAMC2*, *HSPG2*, *THBS1*, *THBS2*, *BGN*, and *VCAN*, was elevated in ADPKD fibroblasts relative to normal kidney fibroblasts (Figure 2B). Together, these findings identify fibroblasts as the dominant source of both fibrous and adhesive ECM in human ADPKD kidneys and demonstrate a marked profibrotic transcriptional program in ADPKD fibroblasts. We therefore next examined whether PFD directly suppresses this profibrotic ECM program in primary human ADPKD myofibroblasts.

### PFD suppresses ECM gene expression in primary human ADPKD renal myofibroblasts.

To validate the ECM expression profile identified by snRNA-seq, we examined ECM gene expression in primary human ADPKD renal myofibroblasts. Consistent with the transcriptomic analysis, these cells expressed the majority of structural fibrous ECM proteins and adhesive ECM glycoproteins identified in ADPKD fibroblasts in vivo (Supplemental Figure 1, A and B; supplemental material available online with this article; https://doi.org/10.1172/jci.insight.207552DS1), confirming that primary ADPKD myofibroblasts retain a robust profibrotic phenotype in culture.

Treatment with PFD broadly suppressed ECM gene expression in ADPKD myofibroblasts. Among structural ECM components, PFD significantly reduced the expression of most collagen genes and ECM-associated proteins examined, with the exceptions of *COL12A1*, *COL15A1*, and lumican (*LUM*), which were not significantly altered (Figure 2, C and D). In contrast, *COL6A2* expression was significantly increased following PFD treatment (Figure 2C). Similarly, PFD significantly decreased the expression of multiple adhesive ECM glycoproteins, whereas nidogen1 (*NID1*) expression remained unchanged (Figure 2D). These findings demonstrate that PFD effectively suppresses the profibrotic ECM transcriptional program in human ADPKD renal myofibroblasts, supporting a direct role for PFD in limiting matrix production by activated stromal cells.

### PFD treatment attenuates renal fibrosis and myofibroblast accumulation in male RC/RC mice.

**Figure 3:**
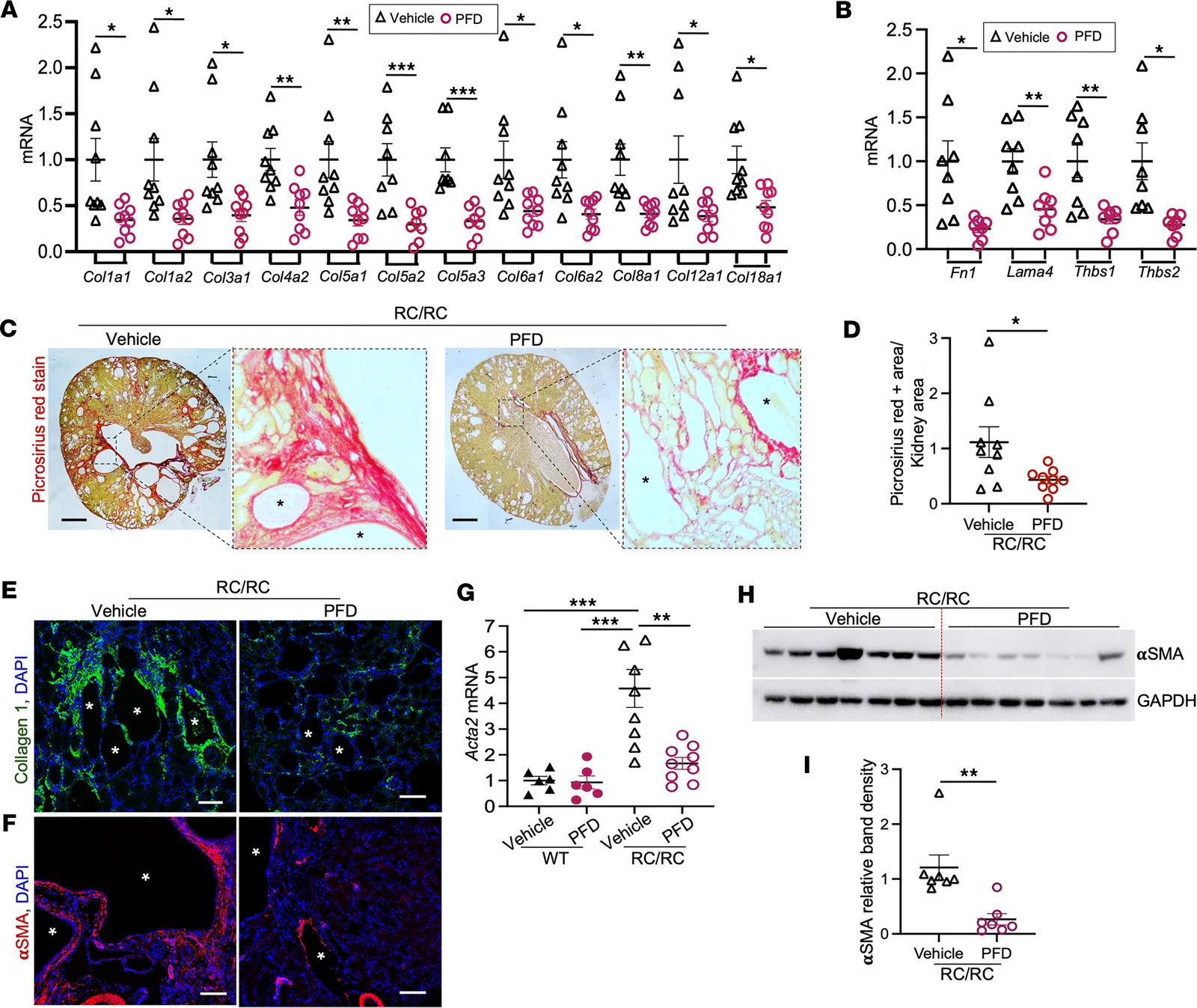
Effect of PFD on fibrosis in male RC/RC mouse kidneys. (**A**) Kidney mRNA levels of structural fibrous ECM proteins and (**B**) cell adhesive-ECM glycoproteins. (**C**) Sirius red staining of mouse kidney tissue (scale bar: 1 mm; original magnification: ×20) and (**D**) quantitation of Sirius red staining. (**E**) Kidney tissue immunostained for collagen 1A (green) and DAPI (nuclei, blue) (scale bar: 100 μm) and (**F**) αSMA (red) (scale bar: 100 μm). (**G**) αSMA mRNA levels relative to 18S in kidney tissue. (**H**) Western blot analysis of the whole-kidney tissue lysate and (**I**) densitometry of Western blots. *P < 0.05, **P < 0.01, ***P < 0.001 by ordinary 1-way ANOVA in **G** and unpaired *t* test with Welch’s correction in **A**, **B**, **D**, and **I**. Stars in **C**, **E**, and **F** represent cysts. In **A**, **B**, **D**, **G**, and **I**, each individual data point represents a biological replicate derived from an independent mouse kidney.

To determine the effect of PFD on renal fibrosis in ADPKD, male RC/RC mice were treated with vehicle or PFD (200 mg/kg, twice daily by oral gavage), from 4 months of age to 6 months of age and sacrificed. PFD treatment significantly reduced expression of ECM genes associated with fibrotic remodeling. Specifically, mRNA levels of multiple collagen genes, including *Col1a1*, *Col1a2*, *Col3a1*, *Col4a2*, *Col5a1*, *Col5a2*, *Col5a3*, *Col6a1*, *Col6a2*, *Col8a1*, *Col12a1*, and *Col18a1*, were significantly reduced in PFD-treated kidneys compared with vehicle-treated control kidneys (Figure 3A). While *Col4a1* and *Col15a1* were also detected, their expression levels were not significantly altered (Supplemental Figure 2A). PFD also significantly reduced expression of several adhesive ECM glycoprotein genes, including *Fn1*, *Lama4*, *Thbs1*, and *Thbs2* (Figure 3B).

Consistent with these transcriptional changes, histological analyses demonstrated a significant reduction in renal fibrosis following PFD treatment. Picrosirius red staining revealed decreased interstitial collagen deposition in PFD-treated RC/RC kidneys compared with vehicle-treated control kidneys (Figure 3, C and D). Immunostaining for collagen I further demonstrated reduced ECM accumulation in PFD-treated kidneys (Figure 3E).

Because myofibroblasts are the principal source of ECM during kidney fibrosis, we next assessed the effects of PFD on myofibroblast abundance. In vehicle-treated RC/RC kidneys, immunostaining revealed αSMA-expressing cells surrounding the cysts. PFD treatment markedly reduced αSMA immunoreactivity (Figure 3F), suggesting decreased myofibroblast accumulation. Quantitative analysis confirmed a significant decrease in both *Acta2* (αSMA) mRNA levels (Figure 3G) and αSMA protein levels (Figure 3, H and I) in PFD-treated mice compared with vehicle-treated controls. Together, these findings demonstrate that PFD attenuates renal fibrosis in RC/RC mice by suppressing profibrotic ECM gene expression, reducing matrix deposition, and limiting myofibroblast accumulation.

### PFD attenuates ECM remodeling and profibrotic signaling in ADPKD.

To investigate the mechanisms underlying the antifibrotic effects of PFD in ADPKD, we examined the expression of genes involved in ECM remodeling, including matrix metalloproteinases (*MMPs*), tissue inhibitors of metalloproteinases (*TIMPs*), a disintegrin and metalloproteinases (*ADAMs*), and matricellular proteins that regulate cell-matrix interactions and myofibroblast activation.

**Figure 4:**
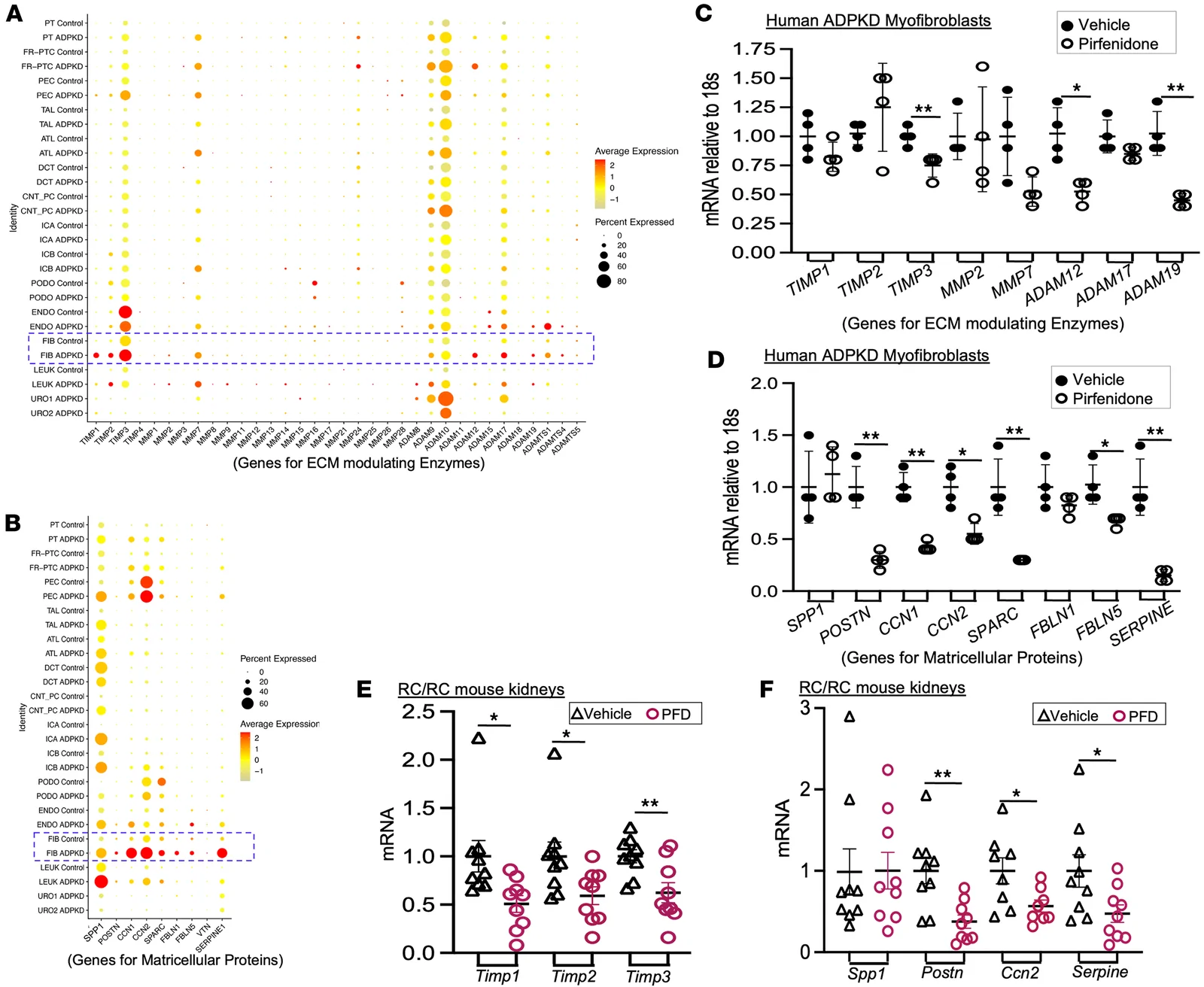
Effect of PFD on ECM modulating enzymes and matricellular proteins in ADPKD. (**A**) Dot plots representing the KIT snRNA-seq dataset, as in Figure 2, show gene expression of ECM-modulating enzymes and (**B**) matricellular proteins. (**C**) In human ADPKD renal myofibroblasts, the effect of PFD (0.5 mg/mL for 24 hours) or vehicle treatment on mRNA levels of ECM modulating enzymes and (**D**) matricellular proteins. (**E**) In RC/RC mouse kidney tissues, mRNA levels of ECM modulating enzymes and (**F**) matricellular proteins. *P < 0.05, **P < 0.01 by unpaired *t* test with Welch’s correction. In **C** and **D**, each individual data point represents a biological replicate (primary myofibroblasts) derived from an independent human ADPKD kidney, and in **E** and **F** each individual data point represents a biological replicate derived from an independent mouse kidney.

Analysis of the KIT snRNA-seq dataset revealed that fibroblasts were a major source of several ECM-remodeling genes in human ADPKD kidneys. Expression of *TIMP1*, *TIMP2*, *TIMP3*, *ADAM12*, and *ADAM17* was enriched in fibroblasts relative to other renal cell populations (Figure 4A). Moreover, *TIMP1*, *TIMP2*, *TIMP3*, *MMP7*, *ADAM10*, *ADAM12*, *ADAM17*, and *ADAMTS1* were expressed at higher levels in ADPKD fibroblasts than in fibroblasts from normal kidneys (Figure 4A). Fibroblasts from ADPKD kidneys also exhibited increased expression of multiple matricellular genes, including osteopontin (*SPP1*), *CCN1, CCN2,* osteonectin (*SPARC*), fibulin1 (*FBLN1*), fibulin5 (*FBLN5*), and *SERPINE1* (Figure 4B), indicating activation of a profibrotic matrix-remodeling program.

To determine whether PFD modulates these fibroblast-associated pathways, we first examined primary cultured human ADPKD myofibroblasts. PFD treatment significantly reduced expression of *TIMP3*, *ADAM12*, and *ADAM19* (Figure 4C), as well as several matricellular genes, including *POSTN*, *CCN1*, *CCN2*, *SPARC*, *FBLN5*, and *SERPINE1* (Figure 4D). Consistent with these in vitro findings, PFD treated RC/RC mouse kidneys exhibited significantly lower expression of *Timp1*, *Timp2*, and *Timp3* (Figure 4E), together with reduced expression of *Postn*, *Ccn2*, and *Serpine1* (Figure 4F), compared with vehicle-treated control kidneys. In contrast, expression of *Mmp2*, *Mmp7*, and *Mmp9* was not significantly altered by PFD treatment (Supplemental Figure 2B).

**Figure 5:**
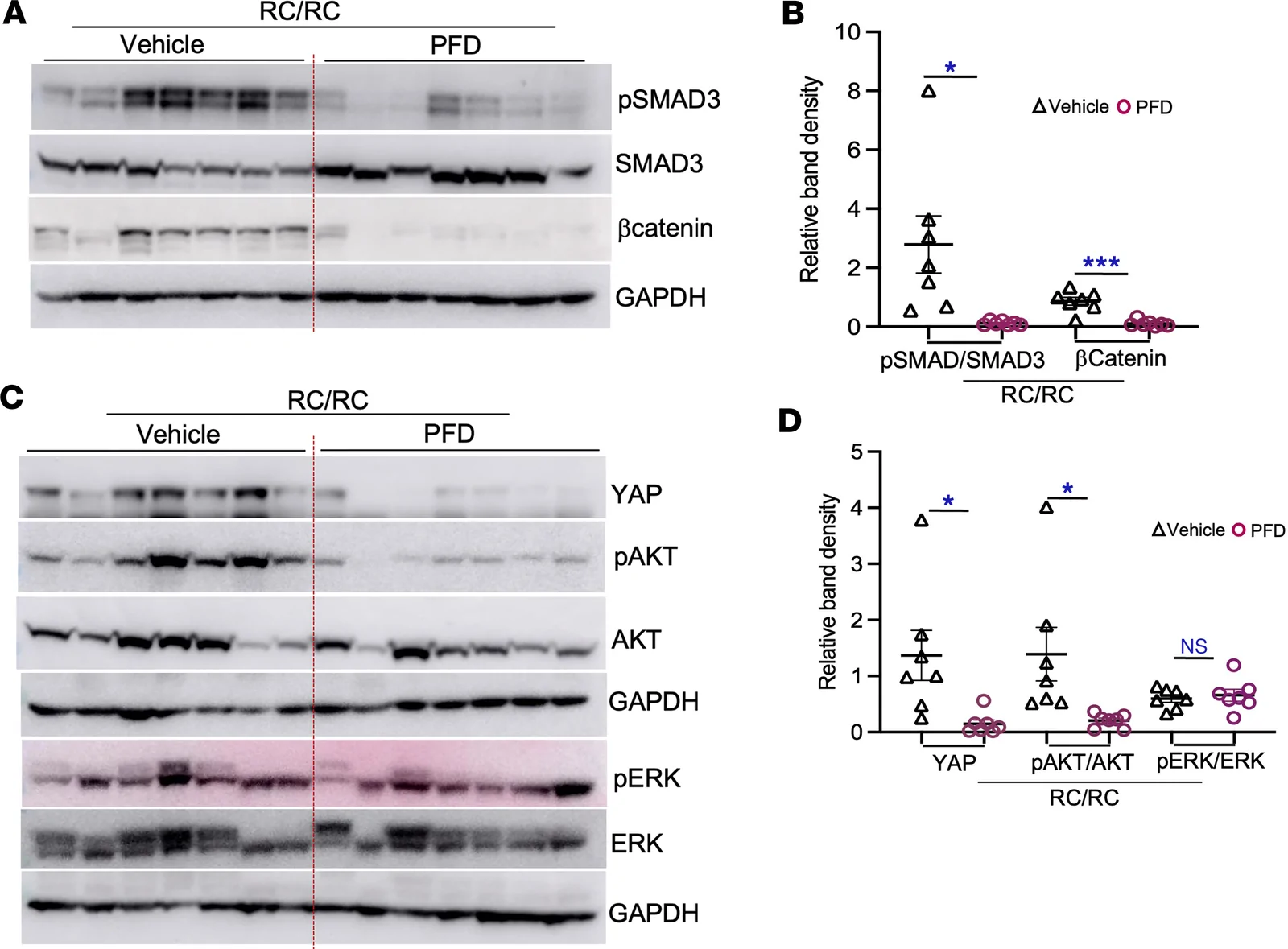
Effect of PFD on profibrotic cell signaling. Western blot analysis of whole-kidney tissue lysate for (**A**) pSMAD3/SMAD3 and β catenin and (**B**) quantification of band density. (**C**) Western blot analysis of whole-kidney tissue lysate for YAP, pAKT/AKT, and pERK/ERK and (**D**) quantification of band density. *P < 0.05, ***P < 0.001 by unpaired *t* test with Welch’s correction. In **B** and **D**, each individual data point represents a biological replicate derived from an independent mouse kidney.

Several of the fibroblast-derived factors suppressed by PFD, including *TIMP3*, *CCN2*, and *THBS1*, have been implicated in activation of TGF-β, AKT, YAP, and β-catenin signaling pathways that promote fibrosis. We therefore assessed whether these downstream pathways were altered in PFD-treated kidneys. PFD treatment significantly reduced TGF-β/SMAD3 and AKT signaling, as evidenced by decreased pSMAD3/SMAD3 and pAKT/AKT ratios, respectively (Figure 5, A–D). PFD also reduced total YAP and β-catenin protein levels (Figure 5, A–D). In contrast, ERK1/2 activity was not significantly altered in PFD-treated RC/RC kidneys (Figure 5, C and D).

To help distinguish treatment-specific effects from disease-related changes, we evaluated WT mice treated with PFD. PFD did not alter αSMA protein levels, pSMAD3/SMAD3 ratios (Supplemental Figure 3, A–C), or expression of *Acta2* (Figure 3G).

Supporting our in vivo findings, TGF-β treatment of cultured NRK-49F rat kidney fibroblasts significantly induced *Acta2* (αSMA) mRNA (Supplemental Figure 4A) and protein expression (Supplemental Figure 4, B and C), which was completely abolished when fibroblasts were treated with PFD (Supplemental Figure 4, A–C). Collectively, these findings demonstrate that PFD suppresses fibroblast-associated ECM remodeling and matricellular signaling programs in ADPKD and is associated with reduced activation of multiple profibrotic pathways in ADPKD kidneys.

### Effect of PFD treatment on cyst growth in RC/RC mouse kidneys.

**Figure 6:**
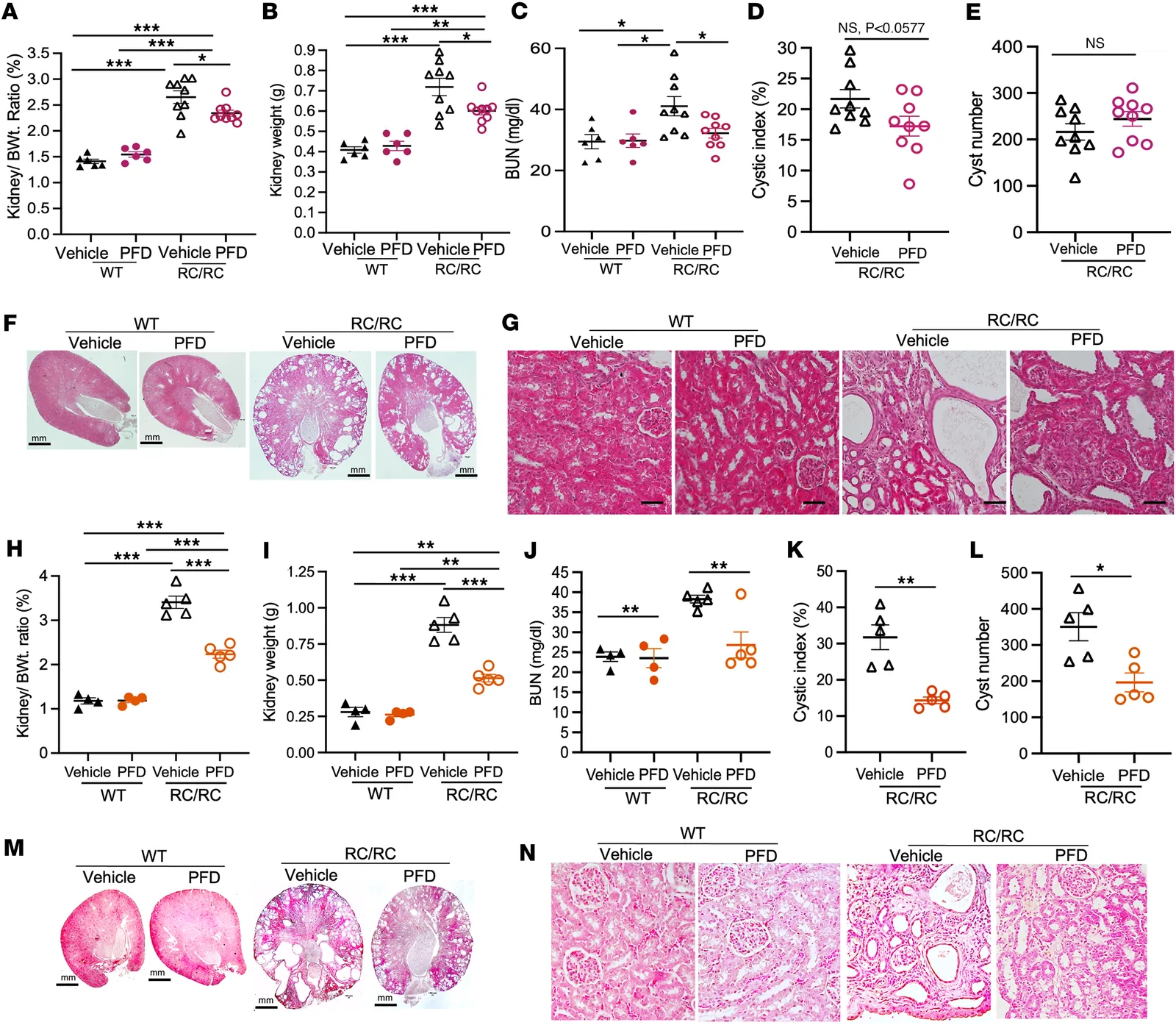
Effect of PFD on cyst growth in male and female RC/RC mice. (**A**–**G**) Male RC/RC mice were treated with vehicle or PFD and then assessed for the following: (**A**) kidney-to-body weight ratios, (**B**) total kidney weight, (**C**) BUN, (**D**) cystic index, and (**E**) cyst number. (**F**) Representative images of H&E staining (scale bar: 1 mm) and (**G**) high-magnification images (scale bar: 100 μm). (**H**–**N**) Female RC/RC mice were treated with vehicle or PFD and then assessed for the following: (**H**) kidney-to-body weight ratios, (**I**) total kidney weight, (**J**) BUN, (**K**) cystic index, and (**L**) cyst number. (**M**) Representative images of H&E staining (scale bar: 1 mm) and (**N**) high-magnification images (scale bar: 100 μm). *P < 0.05, **P < 0.01, ***P < 0.001 by unpaired *t* test with Welch’s correction for **D**, **E**, **K**, and **L** and by ordinary 1-way ANOVA with Tukey’s multiple comparison test in **A**, **B**, **C**, **H**, **I**, and **J**. In **A**–**E** and **H**–**L**, each individual data point represents a biological replicate derived from an independent mouse kidney.

To determine whether the antifibrotic effects of PFD were associated with changes in cystic disease severity, we assessed kidney size, cyst burden, and renal function in RC/RC mice following treatment. In male RC/RC mice, PFD treatment significantly reduced kidney-to-body weight ratio (Figure 6A) and kidney weight (Figure 6B) and improved kidney function, as indicated by lower blood urea nitrogen (BUN) levels (Figure 6C), without affecting overall body weight (Supplemental Figure 5A). Histological analysis demonstrated preservation of renal parenchyma and reduced interstitial expansion in PFD-treated kidneys compared with vehicle-treated control kidneys (Figure 6, F and G). Despite these improvements, PFD did not significantly alter cystic index (Figure 6D) or cyst number (Figure 6E). Expression of the injury markers *Kim1* and *Ngal* also remained unchanged (Supplemental Figure 5, B and C). Plasma alanine transaminase (ALT) and aspartate transaminase (AST) levels showed no significant difference between PFD- and vehicle-treated RC/RC mice, suggesting that liver function was not affected by PFD treatment (Supplemental Figure 5, D and E). PFD treatment had no detectable effects on kidney morphology, kidney size, or BUN levels in WT mice (Figure 6, A, B, and E–G).

Female RC/RC mice exhibited a greater response to PFD treatment. Similar to that in male mice, PFD significantly reduced kidney-to-body weight ratio (Figure 6H) and kidney weight (Figure 6I). In contrast to male mice, however, PFD also significantly reduced cystic index (Figure 6K) and cyst number (Figure 6L). Moreover, expression of the kidney injury markers Kim1 and Ngal was significantly decreased in PFD-treated female RC/RC mice compared with vehicle-treated controls (Supplemental Figure 5, G and H), indicating reduced renal injury. As in the male mice, PFD treatment did not significantly alter plasma ALT and AST levels in female RC/RC mice (Supplemental Figure 5, I and J). PFD treatment in female RC/RC mice also reduced BUN levels (Figure 6J), while preserving renal parenchyma (Figure 6, M and N).

**Figure 7:**
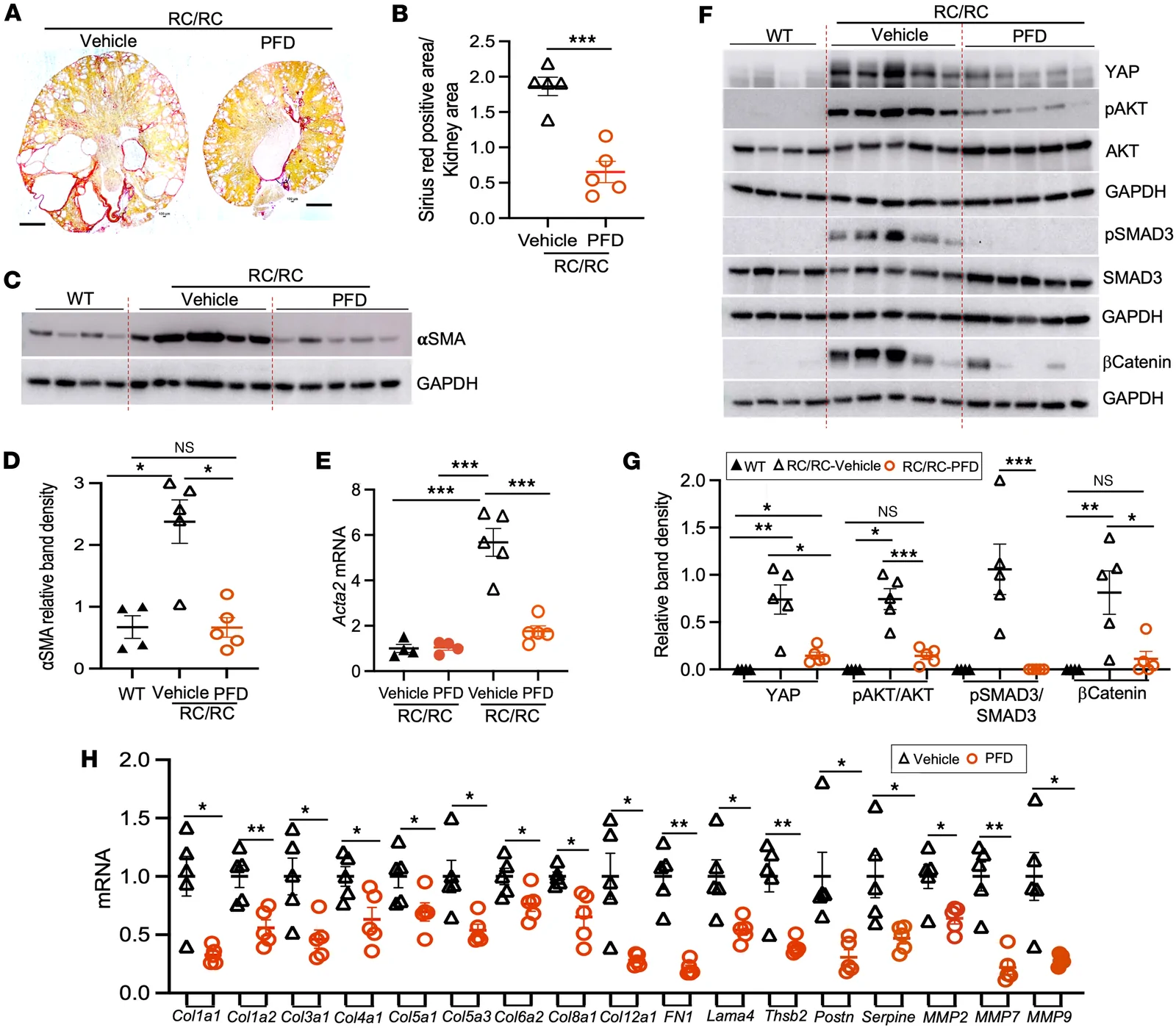
Effect of PFD on fibrosis in female RC/RC mouse kidneys. (**A**) Sirius Red staining of mouse kidney tissue (Scale bar 1mm), and (**B**) Quantitation of Sirius Red staining. (**C**) Western blot analysis of αSMA expression in whole kidney tissue lysate, and (**D**) Quantification of αSMA protein levels (**E**) αSMA mRNA levels relative to 18S in kidney tissue. (**F**) Western blot analysis and (**G**) Densitometry of western blots. (**H**) Kidney mRNA levels of ECM and ECM-related proteins. *P < 0.05, **P < 0.01, ***P < 0.001 by ordinary one-way ANOVA in **D**, **E** and **G**, and unpaired t-test with Welch’s correction in all others. In figures **B**, **D**, **E**,**G** and **H**, each individual datapoint represents a biological replicate derived from an independent mouse kidney.

To determine whether the antifibrotic effects of PFD were similarly maintained in female mice, we evaluated fibrosis and profibrotic signaling pathways. Consistent with the findings in male RC/RC mice, PFD significantly reduced renal fibrosis, as demonstrated by decreased Picrosirius red staining (Figure 7, A and B) and reduced αSMA protein (Figure 7, C and D) and mRNA expression (Figure 7E). PFD also suppressed multiple profibrotic signaling pathways, including TGF-β/SMAD3, AKT, YAP, and β-catenin signaling (Figure 7, F and G). PFD did not alter αSMA protein levels, pSMAD3/SMAD3 ratios (Supplemental Figure 6, A and B), or expression of *Acta2* (Figure 7E) in WT mice. PFD treatment reduced expression of genes encoding structural ECM proteins, adhesive ECM glycoproteins, matricellular proteins, and ECM-remodeling enzymes (Figure 7H), but a subset of ECM and ECM related genes was not significantly affected (Supplemental Figure 7).

Together, these findings demonstrate that PFD improves renal structure and function in RC/RC mice of both sexes and exerts robust antifibrotic effects independent of major changes in cyst burden. However, the additional reduction in cyst growth observed in female mice suggests that suppression of fibrosis-associated pathways may also contribute to limiting cyst progression in a sex-dependent manner.

## Discussion

This study demonstrated that PFD effectively attenuates profibrotic processes in human ADPKD myofibroblasts and reduces renal fibrosis in the RC/RC mouse model of ADPKD. We found that fibroblasts are a major source of ECM in human ADPKD kidneys and that PFD suppresses ECM gene expression, cell proliferation, migration, and contractility in primary human ADPKD renal myofibroblasts in vitro. In RC/RC mice, PFD treatment reduced myofibroblast abundance, ECM accumulation, and profibrotic signaling pathways; decreased kidney enlargement; and improved kidney function in both male and female mice. Together, these findings demonstrate that PFD attenuates fibrosis in ADPKD, at least in part by targeting myofibroblast-driven pathways.

We evaluated the effect of PFD on fibrosis in a mouse model of ADPKD, as well as primary culture human ADPKD kidney myofibroblasts, to better reflect the human disease context. Given the limited characterization of ECM composition in ADPKD kidneys, we analyzed publicly available KIT snRNA-seq datasets, comparing normal and ADPKD human kidneys (20). These analyses identified fibroblasts as the predominant source of multiple structural ECM proteins, adhesive ECM glycoproteins, ECM-remodeling enzymes, and matricellular proteins. Moreover, ADPKD fibroblasts exhibited increased expression of numerous ECM-associated genes compared with fibroblasts from normal kidneys, consistent with a robust profibrotic phenotype. Several of the ECM components enriched in ADPKD fibroblasts have established roles in kidney fibrosis. For example, type I (*COL1A1, COL1A2*) and type III (*COL3A1*) collagens contribute to interstitial fibrosis, ECM stiffening, myofibroblast activation, and vascular remodeling (21). Similarly, type IV collagens (*COL4A1* and *COL4A2*), which are normally confined to basement membranes, have been implicated in pathological ECM remodeling when aberrantly expressed (22). Type V and VI collagens also contribute to ECM remodeling and fibrosis progression (23). In addition, matricellular and matrix-associated proteins, such as periostin, fibrillin-1, fibronectin, thrombospondin1, biglycan, and versican, promote fibrosis through effects on TGF-β signaling, matrix organization, and cell-matrix interactions (24–27). Together, these findings support the concept that fibroblasts are major contributors to the fibrotic microenvironment in ADPKD kidneys.

Importantly, integration of human ADPKD snRNA-seq datasets with functional studies in primary human ADPKD myofibroblasts and the RC/RC mouse model provides convergent evidence that fibroblast-driven ECM production is a prominent feature of ADPKD. The concordance between the human transcriptomic analyses and the experimental responses to PFD strengthens the translational relevance of our findings and supports targeting fibroblast-driven fibrosis as a potential adjunctive therapeutic strategy in ADPKD.

The ECM expression profile identified in the KIT snRNA-seq dataset was validated in primary cultures of human ADPKD renal myofibroblasts. Importantly, PFD treatment suppressed expression of a broad panel of ECM-related genes in these cells. While most ECM and profibrotic genes were consistently downregulated by PFD in both human ADPKD myofibroblasts and RC/RC mouse kidneys, *COL6A2* displayed a divergent response, showing increased expression in cultured human myofibroblasts but not in RC/RC kidneys. This discrepancy may reflect compensatory responses, species-specific differences, or context-dependent effects of antifibrotic therapy and warrants further investigation. Beyond its effects on ECM gene expression, PFD significantly inhibited several fundamental properties of activated myofibroblasts, including proliferation, migration, and matrix contraction. Together, these findings are consistent with suppression of both molecular and functional features of myofibroblast activation in ADPKD.

Consistent with the in vitro findings, PFD treatment significantly reduced the kidney myofibroblast population and ECM accumulation in RC/RC mouse kidneys. PFD treatment was associated with reduced TGF-β/SMAD3 signaling, a pathway widely recognized as a central regulator of fibrosis and a major target of PFD activity in other fibrotic diseases (18). In addition, PFD reduced the expression of ECM-remodeling enzymes and matricellular proteins and was accompanied by suppression of AKT, β-catenin, and YAP signaling pathways, all of which have been implicated in fibrosis and ADPKD progression. Together, the coordinated reduction in ECM production, ECM-remodeling pathways, and profibrotic signaling supports a broad antifibrotic effect of PFD in ADPKD kidneys.

ECM remodeling and ECM stiffening are known to promote cyst growth in ADPKD through aberrant integrin and matricellular signaling (7, 28–30). Similarly, our prior studies show that depletion of αSMA-positive myofibroblasts in RC/RC mice reduces both renal fibrosis and cyst growth (13). Moreover, conditioned media from human ADPKD renal myofibroblasts stimulates proliferation of cyst-lining epithelial cells in vitro, highlighting their contribution to cystogenesis (13). Collectively, these findings suggest that fibrosis contributes not only to progressive tissue remodeling and functional decline, but also to signaling pathways that support cyst expansion.

In the present study, systemic administration of PFD consistently reduced myofibroblast abundance, ECM accumulation, and profibrotic signaling pathways in both male and female RC/RC mice. These antifibrotic effects were accompanied by reductions in kidney-to-body weight ratio and improvements in kidney function in both sexes, indicating an overall attenuation of disease progression. Interestingly, while PFD did not significantly reduce cyst burden in male RC/RC mice, female RC/RC mice exhibited significant reductions in cystic index and cyst number in addition to the antifibrotic response. Importantly, the robust reduction in fibrosis and improvement in kidney function observed in both sexes suggest that the therapeutic benefits of PFD are not solely dependent on effects on cyst growth.

Notably, PFD exerted these beneficial effects without evidence of nephrotoxicity. Kidney injury markers remained stable or were reduced following treatment, and BUN levels improved in both male and female RC/RC mice. PFD also possesses favorable pharmacokinetics in humans, including rapid absorption (time to maximum plasma concentration: 0.33–1 h), half-life of 2–2.5 hours, low interindividual variability, and minimal accumulation with multiple dosing (31). Clinical trials and postmarketing study data for idiopathic pulmonary fibrosis also confirm PFD’s safety and tolerability (32, 33). The most common adverse effects include gastrointestinal symptoms, fatigue, weight loss, and photosensitivity-related rash, which are generally manageable through dose adjustment (34). We previously reported that nintedanib, another FDA-approved antifibrotic agent used for idiopathic pulmonary fibrosis, reduces kidney fibrosis and cyst growth while preserving kidney function in ADPKD mice models (35). Although nintedanib may exert broader biological effects through inhibition of multiple tyrosine kinases, it is also associated with a higher incidence of gastrointestinal adverse events compared with PFD (36).

Several limitations should be considered when interpreting these findings. First, many of the signaling analyses were performed using whole-kidney lysates, which precludes definitive assignment of pathway-specific effects to individual renal cell populations. Although our in vitro studies support a direct effect of PFD on ADPKD myofibroblasts, indirect effects on cyst-lining epithelial cells, inflammatory cells, or other stromal populations may also contribute to the observed in vivo responses. Second, although PFD significantly reduced fibrosis and improved kidney function in the present study, and reduced cyst burden in female RC/RC mice, future long-term studies will be required to determine the durability of these effects, evaluate long-term safety, and assess whether sustained treatment can further modify disease progression in ADPKD. Finally, the basis for the greater reduction in cyst burden observed in female mice remains unclear and warrants future investigation into potential sex-specific differences in disease biology and responsiveness to antifibrotic therapy.

Given that current treatments for ADPKD, such as tolvaptan, primarily target pathways associated with cyst growth, antifibrotic therapies may provide a complementary approach by addressing progressive fibrotic remodeling. In the present study, PFD consistently reduced renal fibrosis and improved kidney function in both male and female RC/RC mice, while reducing cyst burden in female mice. Collectively, these findings support the concept that targeting fibroblast-driven fibrosis represents a promising adjunctive strategy for slowing disease progression in ADPKD.

## Methods

### In vivo study

#### Sex as a biological variable.

Both male and female mice were used in this study.

#### ADPKD mouse model.

The RC/RC mouse (37, 38) is a slow progressing, adult, orthologous model of ADPKD carrying a temperature-sensitive folding hypomorphic mutation (R3277C) in the Pkd1 gene. Mice are on pure BALB/c background and inbred. Male WT and RC/RC mouse littermates were treated with vehicle (5% DMSO+1% hydroxymethyl cellulose) or PFD (HY-B0673, MedChemExpress) (200 mg/kg body weight, twice daily) by oral gavage between 8–9 AM and 4–5 PM, 6 days a week, from 4 to 6 months of age and sacrificed at 6 months of age. The selected dose was based on published preclinical studies (39–43), and pilot dose-finding experiments demonstrating antifibrotic efficacy without adverse effects on body weight. Power analysis conducted based our previous study (35) determined that using 8 mice will provide 80% statistical power to detect a 23% reduction in kidney-to-body weight ratio in RC/RC mice treated with drug compared with controls (significance level [α] 0.05). Mice from each litter were assigned to study groups as they reached 4 months of age. No randomization was used. We selected 4 months of age as the start of the treatment because at this age, both male and female RC/RC mice showed significant increase in kidney-to-body weight ratio and renal aSMA mRNA levels compared with WT littermate mice (Supplemental Figure 8).

Investigators performing the study, and analyses were blinded to the identity of the mice. Mice were housed in a temperature controlled environment in a 12-hour-light/12-hour-dark cycle. Experimental mice were placed on the same rack. All mice were sacrificed between 11:30 AM and 12:30 PM. Blood was collected and plasma isolated. Kidneys were weighed and flash frozen or fixed in 4% paraformaldehyde. No adverse effects were observed, and no mice or data points were excluded. Studies were approved by University of Kansas IACUC committee and ARRIVE guidelines (44) were followed.

#### H&E staining and quantification of cysts.

H&E staining was performed on 5 μm thick kidney tissue sections and imaged using Nikon 80i upright microscope. Cyst number and cystic index (cyst area/total area per kidney tissue section) were quantified using ImageJ (NIH) by an observer blinded to the sample’s identity (45).

#### Picrosirius red staining and quantification of tissue fibrosis.

Formalin-fixed, paraffin-embedded mouse kidney sections were stained with Picrosirius red (46) according to manufacturer’s instructions (ScyTek Laboratories, PSR-2) to detect collagen fibers. Kidney fibrosis was quantitatively evaluated using polarized light microscopy in combination with image analysis software (QuPath). Collagen accumulation was determined by calculating the proportion of birefringent staining relative to the total renal tissue area.

#### BUN levels.

Plasma BUN levels was measured using the commercially available QuantiChrom Urea Assay Kit (DIUR-100, BioAssay Systems) (47).

#### Western blot.

Kidneys were homogenized in SDS Laemmli buffer and run in 10% SDS-polyacrylamide agarose electrophoresis gels (48). Primary antibodies for αSMA (ab5694; Abcam); pERK1/2 (9102S), ERK1/2 (9101S), β-catenin (9582S), pAKT (4060S), AKT (4691T), pSMAD3 (9520T), and SMAD3 (9513S) from Cell Signaling; YAP (SC-101199); GAPDH (SC-32233, Santa Cruz Biotechnology Inc.); and anti-mouse (P0447) and anti-rabbit (P0448) secondary antibodies from Dako (Santa Clara, CA, USA) were used. Immunoreactive proteins were detected using ECL reagent (Amersham, GE Healthcare).

#### Immunofluorescence staining.

Fixed and paraffin-embedded tissues sections were processed as previously described (49). Primary antibodies αSMA (ab5694) from Abcam and Collagen Type-1a (203002 from MD Bioproducts) were applied followed by incubation with secondary antibodies anti-Rabbit IgG Alexa fluor 488 and anti-Goat IgG Alexa fluor 594 from Invitrogen. After incubation, tissue sections were washed, stained with DAPI, and mounted using Flour-G (Invitrogen). Images were captured using a Nikon 90i upright microscope.

#### Quantitative real time PCR.

RNA was isolated from whole-kidney lysate using the TRIzol method (Invitrogen, Thermo Fischer Scientific), and cDNA prepared with the High-capacity cDNA reverse transcription kit (4368814, Applied Biosystems). SYBR Green PCR master mix (A25742, Applied Biosystems) was used for QRT-PCR (50). Supplemental Table 1 shows the primer sequences.

#### Liver function tests.

Serum ALT and AST levels were measured using commercially available Pointe Scientific assay kits (23-666-087and 23-666-121, respectively, Pointe Scientific) following the manufacture’s instructions.

### In vitro studies

#### Primary culture human ADPKD myofibroblasts.

Cells were isolated from human ADPKD kidney tissues obtained from the University of Kansas PKD Biomarkers and Biomedical core (35, 51). Cells were used in their first passage and grown in DMEM:F12 media with 10% FBS and 1% penicillin/streptomycin (Cytiva, Hyclone Laboratories). Mycoplasma contamination was monitored using DAPI staining and confirmed using a PCR-based mycoplasma testing kit (ab289834, Abcam Inc.).

#### NRK-49F rat kidney fibroblasts.

NRK-49F rat kidney fibroblasts (CRL-1570, ATCC) were grown in DMEM medium with 5% FBS and 1% penicillin/streptomycin.

#### Cell viability and cell proliferation assays.

3-(4,5-dimethylthiazol-2-yl)-2,5-diphenyltetrazolium bromide (MTT) assay was conducted to assess cell viability (38). To measure cell proliferation by BrdU incorporation assay (38), human ADPKD kidney myofibroblasts grown on coverslips were serum starved overnight followed by release into media containing 0.2% FBS with PFD or vehicle. After 24 hours, cells were incubated with 3 μg/mL BrdU (10280879001, MilliporeSigma) for 3 hours, and cell proliferation was measured as BRDU/DAPI expressed as a percentage.

#### Migration assay.

Confluent monolayers of human ADPKD kidney myofibroblasts were treated with 5 μg/mL mitomycin c (M7949; Sigma-Aldrich) for 2 hours and washed twice with PBS; a single scratch (wound) was created manually across the monolayer using a sterile pipette tip; and cell debris washed off using PBS. The cells were treated with vehicle or PFD. The wound was imaged at regular intervals using a phase-contrast microscope (Nikon ECLIPSE TE2000-U, Nikon) until the wounds in any one study group closed 100%, and wound closure was quantified (52).

#### Gel contractility assay.

Human ADPKD myofibroblasts were trypsinized and resuspended in complete medium and mixed with rat-tail collagen type-1 (pH 7.4) (354236, Corning). 500 μL of collagen/cell mixture containing 1.5 × 10^5^ cells was dispensed into 24-well cell culture plates coated with 0.2% BSA. The mixture was incubated to polymerize at 37ºC in a CO_2_ incubator for 1 hour. After polymerization, the gels were gently detached from the sides and were incubated with serum free medium. The gels were imaged at different time points, and the gel area was calculated using ImageJ software.

#### scRNA-seq.

The snRNA-seq data were obtained from the KIT database by Y. Muto et al. (20) (GEO GSE185948; https://humphreyslab.com/SingleCell/search.php). The bioinformatics analysis of this data was performed in line with the description given by the authors in their Methods. We recreated the exact R environment used by the authors in order to obtain results comparable to their’s for genes of our interest presented in the dot plots.

#### Statistics.

Values are expressed as mean ± SEM for in vivo studies and mean ± SD for in vitro studies. The data were analyzed by 2-tailed unpaired *t* test with Welch’s correction or 1-way ANOVA followed by Tukey`s multiple comparisons test. *P* ≤ 0.05 was considered statistically significant. Analysis was done using GraphPad Prism software version 10.

#### Study approval.

Mouse studies were approved by University of Kansas IACUC committee, and ARRIVE guidelines (44) were followed.

#### Data availability.

All in vivo studies using mouse models were performed and tissues were analyzed at the University of Kansas Medical Center. All data that were generated are provided in the figures in the main text or in supplemental material. Values for all data points in graphs are reported in the Supporting Data Value file.

All requests for data will be processed based on institutional policies for noncommercial research purposes and shared. Data sharing could require a data transfer agreement, as determined by the University of Kansas Medical Center’s legal department.

## Author contributions

RR conceptualized and designed studies, analyzed results, and wrote the paper. VR performed experiments, analyzed results, and wrote parts of the paper. AJ, MMV, and HY performed some studies and analyzed results. SG performed bioinformatics analysis and generated the dot plots from the publically available KIT database. DPW conceptualized some studies and reviewed the paper. All authors read, edited and approved the paper.

## Conflict of interest

The authors have declared that no conflict of interest exists.

## Funding support

This work is the result of NIH funding, in whole or in part, and is subject to the NIH Public Access Policy. Through acceptance of this federal funding, the NIH has been given a right to make the work publicly available in PubMed Central.

- NIH grant R01DK135308-01 to RR.
- US Department of Veterans Affairs Merit Review Award # I01RD001368-01A1, Biomedical Laboratory Research and Development Service to RR.
- American Heart Association Postdoctoral fellowship grant 26POST1562710 toVR.

## Supplementary Material

Supplemental data

Unedited blot and gel images

Supporting data values

## Version 1 09/22/2026

Electronic publication
