# Supplementary material for: Pirfenidone treatment attenuates fibrosis in autosomal dominant polycystic kidney disease
Source: JCI Insight. 2026 Sep 22;11(18):e207552. doi: 10.1172/jci.insight.207552 (PMC13596711; doi:10.1172/jci.insight.207552)
Supplement: Supplementary file 1 [file jciinsight-11-207552-s309.pdf]

Supplemental Figures

Supplemental Fig. 1

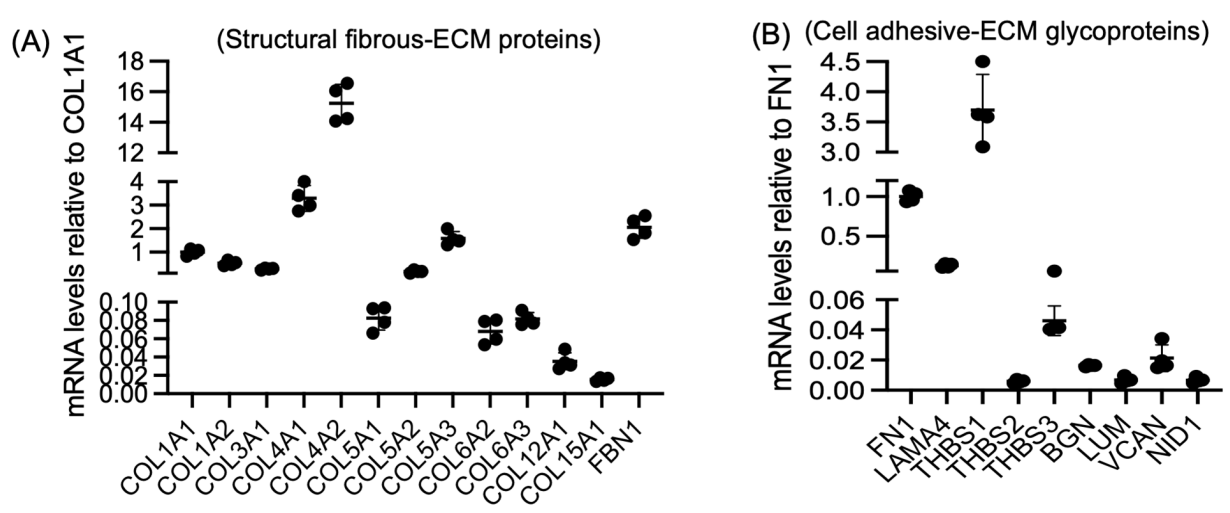

Supplemental Figure 1: (**A**) In primary culture human ADPKD myofibroblasts, relative gene expression of structural fibrous ECM proteins, (**B**) cell adhesive ECM-glycoproteins. Each individual data point represents a biological replicate (cells from individual human ADPKD kidneys).

Supplemental Fig. 2

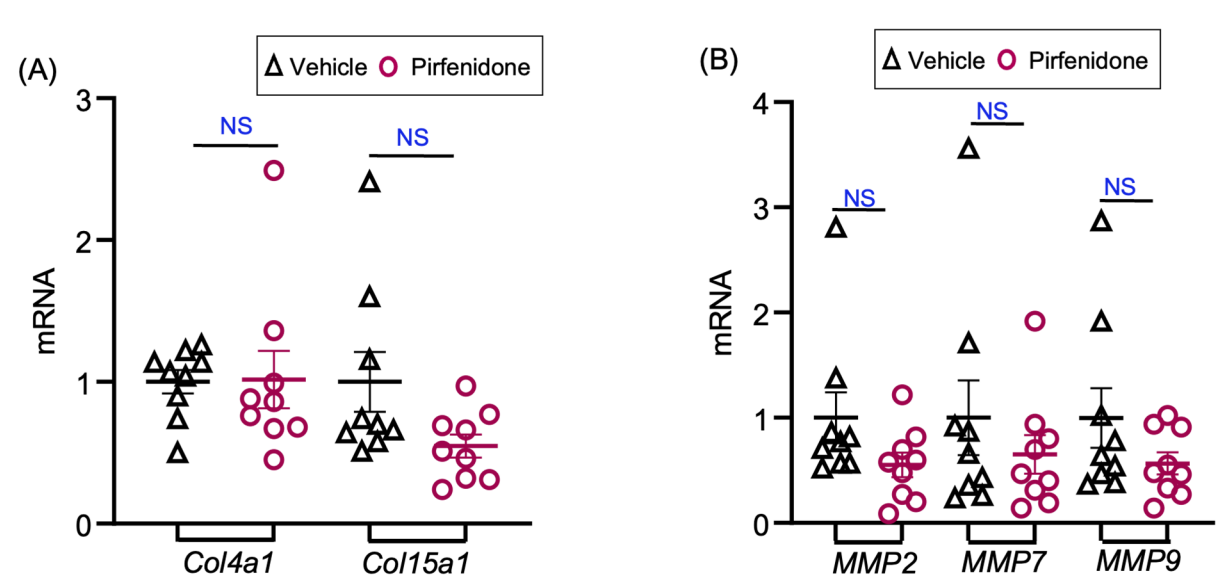

Supplemental Figure 2: Supplemental Figure 2: mRNA levels relative to 18S for (**A**) Col4a1 and Col15a1, and (**B**) MMPs in RC/RC mouse renal tissues.Each each individual datapoint represents a biological replicate derived from an independent mouse kidney.

Supplemental Fig. 3

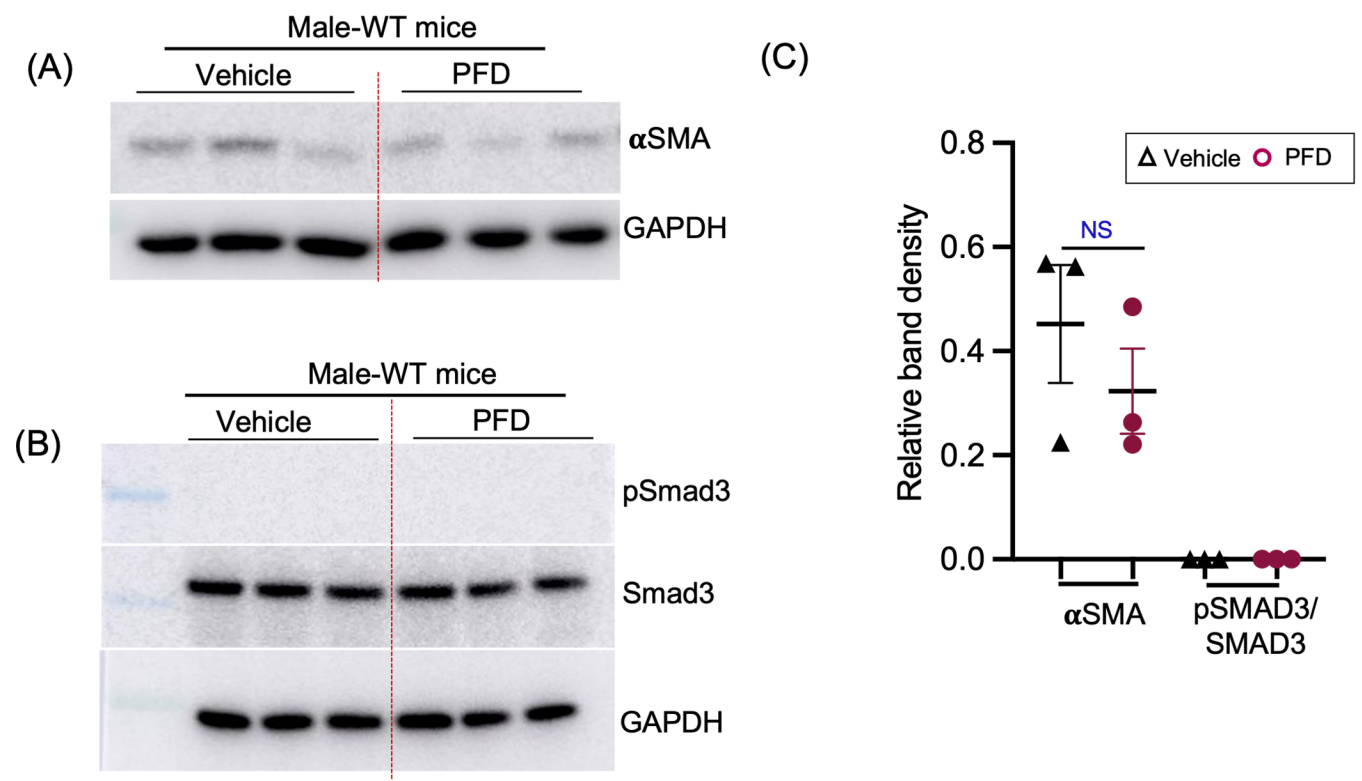

Supplemental Figure 3: Western blot analysis of whole kidney tissue lysate from WT male mice for, (**A**) αSMA (**B**) pSMAD3/SMAD3 and (**C**) Quantification of band density.Each individual datapoint represents a biological replicate derived from an independent mouse kidney.

Supplemental Fig. 4

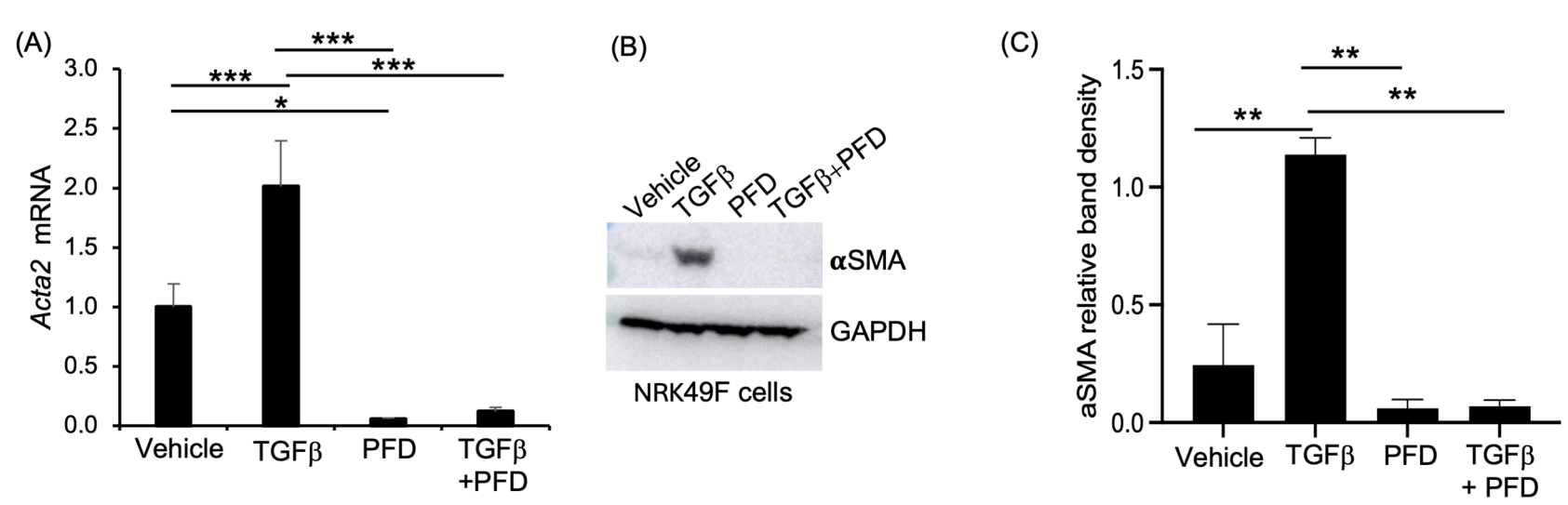

Supplemental Figure 4: (**A**) mRNA levels relative to 18S for αSMA in NRK-49F cells treated with TGFβ and pirfenidone for 24h. (**B**) Western blot analysis of NRK-49F cells treated with TGFβ (2ng/ml) and pirfenidone (0.5mg/ml) for 24h.(**C**) quantification of band density. *P<0.05, **P<0.01, ***P<0.001 by two-tailed unpaired t-test with Welch's correction.

Supplemental Fig. 5

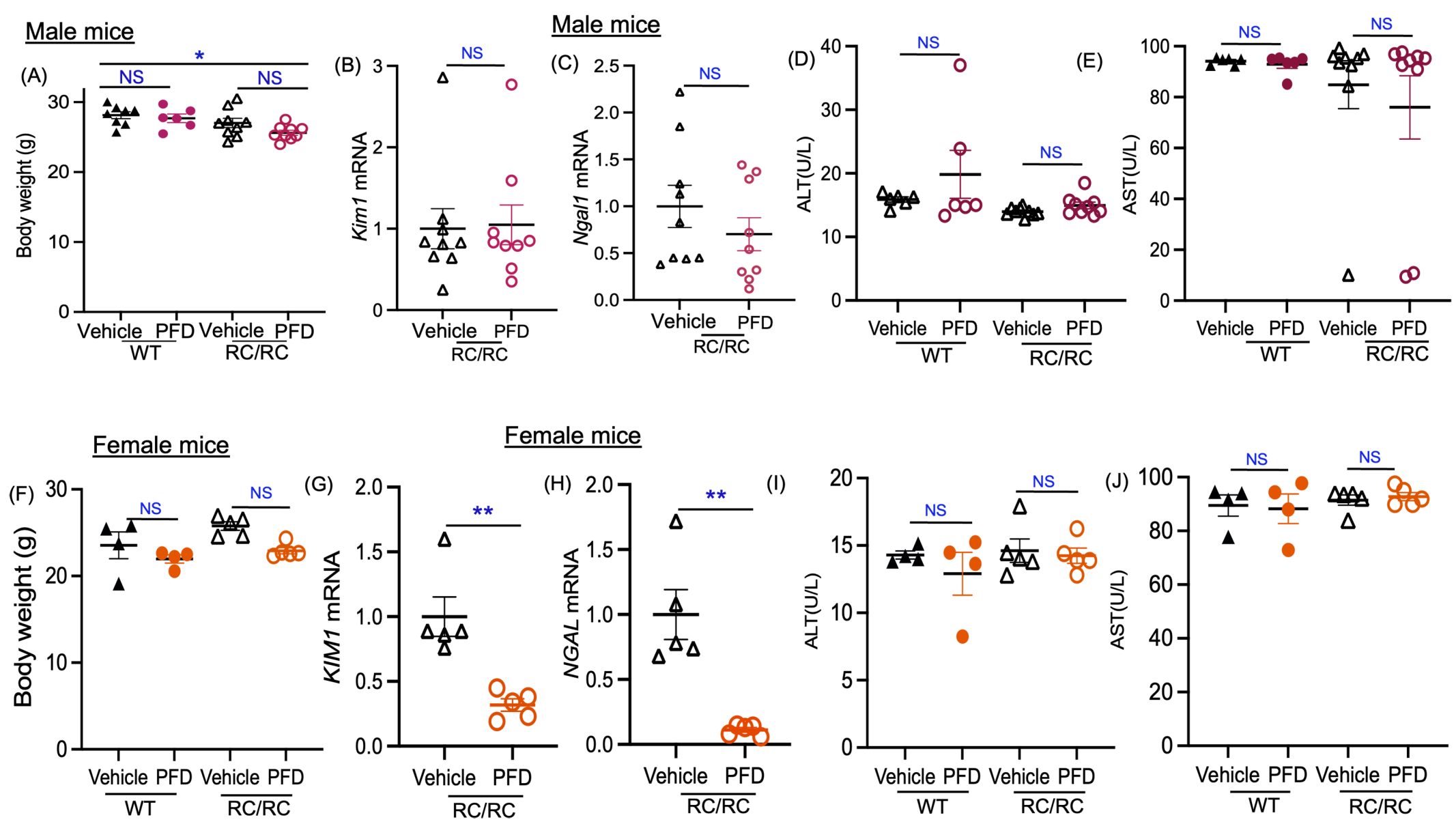

Supplemental Figure 5: (**A**) Body weight of male mice. (**B**) mRNA levels relative to 18S for *Kim1* and (**C**) *Ngal* in male mouse kidey tissue. (**D**) Serum ALT (U/L) in male mice (E) Serum AST (U/L) in male mice.(**F**) Body weight of female mice. (**G**) mRNA levels relative to 18S for *Kim1* and (**H**) *Ngal* in female mouse kidey tissue. (**I**) Serum ALT (U/L) in female (J) Serum AST (U/L) in female mice.Each individual datapoint represents a biological replicate derived from an independent mouse kidney.

Supplemental Fig. 6

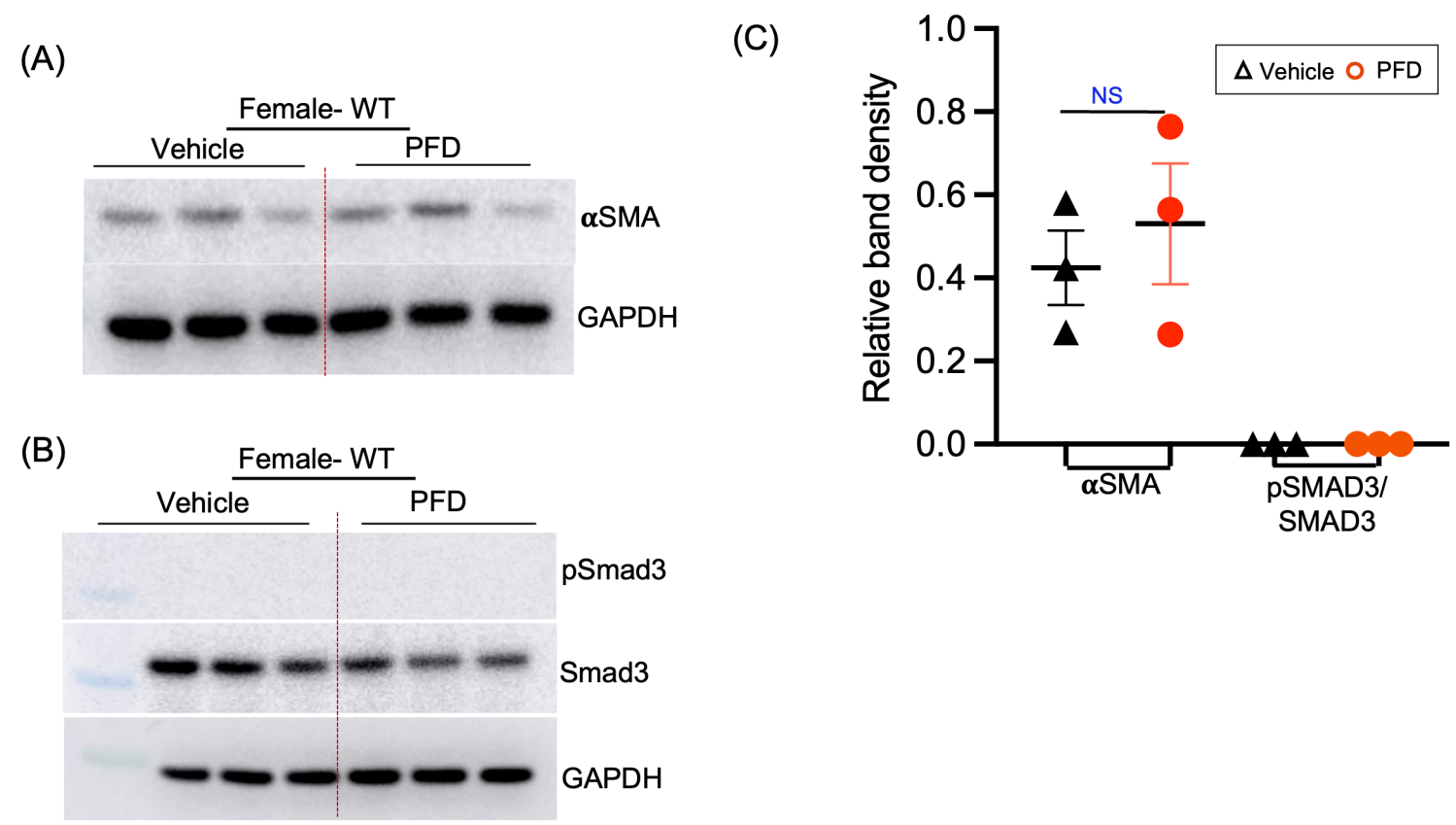

Supplemental Figure 6: Western blot analysis of whole kidney tissue lysate from WT female mice for, (**A**) αSMA (**B**) pSMAD3/SMAD3 and (**C**) Quantification of band density.Each individual datapoint represents a biological replicate derived from an independent mouse kidney.

Supplemental Fig. 7

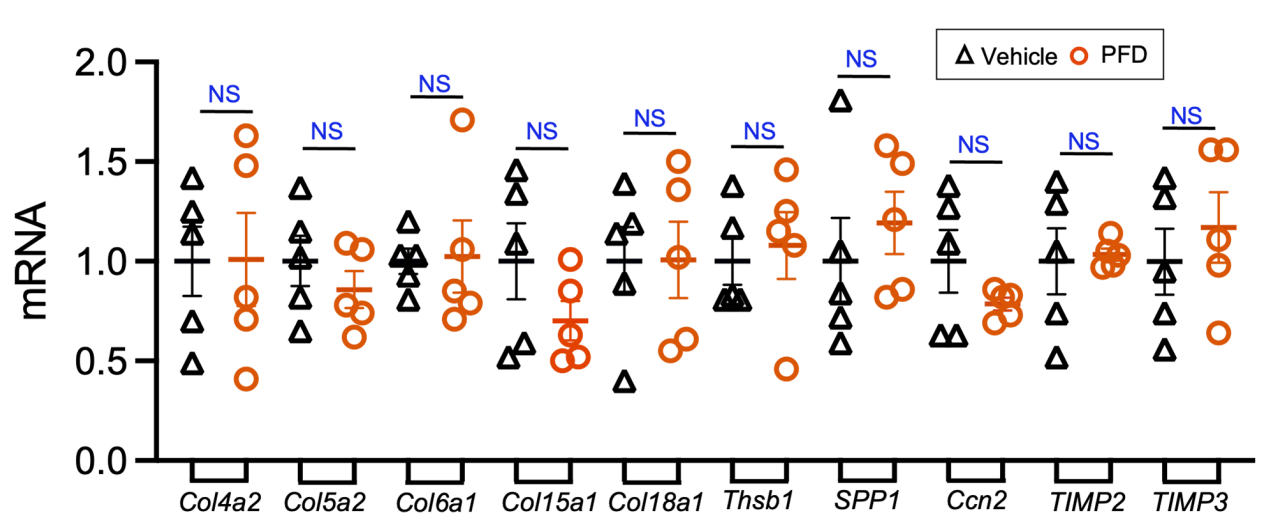

Supplemental Figure 7: mRNA levels relative to 18S for Col4a2, Col5a2, Col6a1, Col15a, Col18a1, Thsb1, SPP1,SPP1,Ccn2,TIMP2 and TIMP3 in female RC/RC mouse renal tissues.Each each individual datapoint represents a biological replicate derived from an independent mouse kidney.

4 months old mice

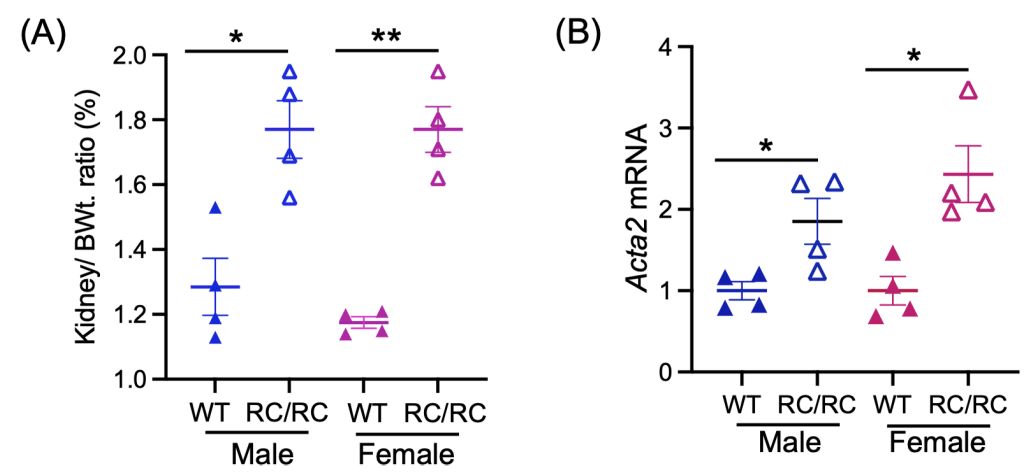

Supplemental Figure 8: Male and female RC/RC mice at 4 months of age. (**A**) Kidney to body weight ratio, (**B**) mRNA levels relative to 18S for *αSMA*. P<0.05 by two-tailed unpaired t-test with Welch's correction. Each individual datapoint represents a biological replicate derived from an independent mouse kidney.

Supplemental Table-1

Human primer sequence used for QRT-PCR

	Gene Name	Primer Forward (F) and Reverse (R)
1.	*MMP-2*	F-AGCGAGTGGATGCCGCCTTTAA<br>R-CATTCCAGGCATCTGCGATGAG
2.	*MMP-7*	F-TCGGAGGAGATGCTCACTTCGA<br>R- GGATCAGAGGAATGTCCCATACC
3.	*LAMA-4*	F-GAGATGACTCTCTGCTGGACCT<br>R-AGTTCCAGGCAGCCAACAAAGC
4.	*TIMP-1*	F-GGAGAGTGTCTGCGGATACTTC<br>R-GCAGGTAGTGATGTGCAAGAGTC
5.	*TIMP-2*	F-ACCCTCTGTGACTTCATCGTGC<br>R-GGAGATGTAGCACGGGATCATG
6.	*TIMP-3*	F-TACCGAGGCTTCACCAAGATGC<br>R-CATCTTGCCATCATAGACGCGAC
8.	*BGN*	F-TTGAACCTGGAGCCTTCGATGG<br>R-TTGGAGTAGCGAAGCAGGTCCT
9.	*LUM*	F-AACATACCAACTGTCAATGAAAACC<br>R-TGCCATCCAAACGCAAATGCTTG
10.	*FBN1*	F-GGATACACAGGTGATGGCTTCAC<br>R-GTCGCATTCACAGCGGTATCCT
11.	*VCAN*	F-TTGGACCTCAGGCGCTTTCTAC<br>R-GGATGACCAATTACACTCAAATCAC
12.	*NID1*	F-ACATTGAGCCCTACACGGAGCT<br>R-GCCACTGGTAAGTGTAGATGCG
13.	*ADAM12*	F-ATGGCATCTGCCAGACTCACGA<br>R-GGAACTCTTCGAGACTTTGCCAC
14.	*ADAM17*	F-AACAGCGACTGCACGTTGAAGG<br>R-CTGTGCAGTAGGACACGCCTTT
15.	*ADAM19*	F-CGAGAAGGTGAATGTGGCAGGA<br>R-AGCTCTGACACTGGATCTTCCC
16.	*THBS1*	F-GCTGGAAATGTGGTGCTTGTCC<br>R-CTCCATTGTGGTTGAAGCAGGC
17.	*THBS2*	F-CAGTCTGAGCAAGTGTGACACC<br>R-TTGCAGAGACGGATGCGTGTGA
18.	*THBS3*	F-GGAAGGAGATGCCTGTGACAAC<br>R-GGTAGGATTGCTCATTTCAGGGC
19.	*SPARC*	F-TGCCTGATGAGACAGAGGTGGT<br>R-CTTCGGTTTCCTCTGCACCATC
20.	*FBLN1*	F-GTGGCTACCATCTCAACGAGGA

		R-CTTGCATTCGCAGCGGAAACTG
21.	*FBLN5*	F-CTGCTGGATGACAACCGAAGCT<br>R-GATAAGGCTCCTCACAGCGGAT
22.	*CCN1*	F-GGAAAAGGCAGCTCACTGAAGC<br>R-GGAGATACCAGTTCCACAGGTC
23.	*CCN2*	F-CTTGCGAAGCTGACCTGGAAGA<br>R-CCGTCGGTACATACTCCACAGA
24.	*COL1a1*	F-TGACGTGATCTGTGACGAGAC<br>R-GGTTTCTTGGTCGGTGGGT
25.	*COL1a2*	F-CCTGGTGCTAAAGGAGAAAGAGG<br>R-ATCACCACGACTTCCAGCAGGA
26.	*COL3a1*	F- TGG TCT GCA AGG AAT GCC TGGA<br>R- TCT TTC CCT GGG ACA CCA TCAG
27.	*COL4a1*	F-TGTTGACGGCTTACCTGGAGAC<br>R-GGTAGACCAACTCCAGGCTCTC
28.	*COL4a2*	F-GGATAACAGGCGTGACTGGAGT<br>R-CTTTGCCACCAGGCAGTCCAAT
29.	*COL5a1*	F- GGAGATGATGGTCCCAAAGGCA<br>R- CCATCATCTCCTTTGTCACCAGG
30.	*COL5a2*	F- CAGGCTCCATAGGAATCAGAGG<br>R- CCAGCATTTCCTGCTTCTCCAG
31.	*COL5a3*	F-GAGAGGAGAACTGGGCTTCCAA<br>R-TAGAGGTCCCACTTCTCCTGTC
32.	*COL6a2*	F-CGTGGAGACTCAGGACAGCCA<br>R-CCTTTCAAGCCAAAGTCGCCTC
33.	*COL6a3*	F-CCTGGTGTAACTGATGCTGCCA<br>R-AAGATGGCGTCCACCTTGGACT
34.	*COL12a1*	F-CAGTGCCTGTAGTCAGCCTGAA<br>R-GGTCTTGTTGGCTCTGTGTCCT
35.	*COL15a1*	F- GGTGACACTGGTTTACCTGGCT<br>R-GCCTTTCCAGAGGAATGTCCTC
36.	*POSTN*	F- TGCCCAGCAGTTTTGCCCAT<br>R- CGTTGCTCTCCAAACCTCTA
37.	*SERPINE1*	F- CTCATCAGCCACTGGAAAGGCA<br>R- GACTCGTGAAGTCAGCCTGAAAC
38.	*FBLN1*	F- GTGGCTACCATCTCAACGAGGA<br>R- CTTGCATTCGCAGCGGAAACTG
39.	*FBLN5*	F- CTGCTGGATGACAACCGAAGCT<br>R- GATAAGGCTCCTCACAGCGGAT
40.	*18s*	F-ACCGCGGTTCTATTTTGTTG<br>R- CCCTCTTAATCATGGCCTCA

Mouse primer sequence used for QRT-PCR

	Gene Name	Primer Forward (F) and Reverse (R)
1.	*Adam12*	F-TGCTACAACGGCATCTGCCAGA<br>R-GCTCTTGGAGTCTTTGCCACAG
2.	*Adam17*	F-TGTGAGCGGTGACCACGAGAAT<br>R-TTCATCCACCCTGGAGTTGCCA
3.	*Adam19*	F-GTGCCTCACTTACCAGGAACAG<br>R-GGACTGCACTTCCTGTATTGGC
4.	*Thbs1*	F-GGTAGCTGGAAATGTGGTGCGT<br>R-GCACCGATGTTCTCCGTTGTGA
5.	*Thbs2*	F-GTATGGAGGGAAGGACTGTGTC<br>R-ACTTGGCTCCAGGAAAACACGG
6.	*SPARC*	F-CACCTGGACTACATCGGACCAT<br>R-CTGCTTCTCAGTGAGGAGGTTG
7.	*Lama4*	F-CAGTTTGTCCTCTACCTCGGAAG<br>R-CTCACAGGCTTGGAATCCAGGA
8.	*Bgn*	F-TGAACCAGGAGCCTTTGATGGC<br>R-GTCCTCCAACTCAATAGCCTGG
9.	*Vcan*	F-GGACCAAGTTCCACCCTGACAT<br>R-CTTCACTGCAAGGTTCCTCTTCT
10.	*Col1a1*	F - AGACATGTTCAGCTTTGTGGAC<br>R - GCA GCT GAC TTC AGG GATG
11.	*Col1a2*	F-TTCTGTGGGTCCTGCTGGGAAA<br>R-TTGTCACCTCGGATGCCTTGAG
12.	*Col3a1*	F - TCC CCT GGA ATC TGT GAA TC<br>R - TGA GTC GAA TTG GGG AGA AT
13.	*Col4a1*	F-CGGGTGTGAAAAGACCTATCGG<br>R-CTGGCATTCCTCTGACGCCTTT
14.	*Col4a2*	F-CGGGTGTGAAAAGACCTATCGG<br>R-CTGGCATTCCTCTGACGCCTTT
15.	*Col5a1*	F-AGATGGCATCCGAGGTCTGAAG<br>R-GACCTTCAGGACCATCTTCTCC
16.	*Col5a3*	F-GGCAAAGATGGTATTCCAGGACC<br>R-TGCTTCCTTTGTGACCAGGCATC
17.	*Col5a2*	F-GTGGCATAGGAGAGAAAGGTGC<br>R-GCCAACTAAGCCTCTAGGACCA
18.	*Col6a1*	F-GACACCTCTCAGTGTGCTCTGT<br>R-GCGATAAGCCTTGGCAGGAAATG
19.	*Col6a3*	F-CCTGGTGTAACTGATGCTGCCA<br>R-AAGATGGCGTCCACCTTGGACT
20.	*Col8a1*	F-GGAAATCCCACCTGTGCCAAGA<br>R-TCCTCTTGGTCCAGGTTCTCCA
21.	*Col12a1*	F-CAGCACCATGAATGTCGTCTGG<br>R-GGTCTTTGAGGATAGTGCTGGC
22.	*Col15a1*	F-ACACCCACAGTGACTCCCAAGA<br>R-TCCTCATTGCCCACGATGTCTC
23.	*Col18a1*	F-GTGACACTGGACCTCAAGGCTT<br>R-TTGTCTGAAGGAGGGTCCTGGT
24.	*CCN1*	F-GTGAAGTGCGTCCTTGTGGACA<br>R-CTTGACACTGGAGCATCCTGCA
25.	*CCN2*	F - GTGCCAGAACGCACACTG<br>R - CCCCGGTTACACTCCAAA
26.	*aSMA*	F - TCAGGGAGTAATGGTTGGAATG

		R - GGTGATGATGCCGTGTTCTA
27.	*18s*	F - GTAACCCGTTGAACCCGA<br>R - CCATCCAATCGGTAGTAGCG
28.	*Timp1*	F - CAAGGATGGACTCCTGGCACAT<br>R - TACTCGCCATCAGCGTTCCCAT
29.	*Timp2*	F - AGCCAAAGCAGTGAGCGAGAAG<br>R - GCCGTGTAGATAAACTCGATGTC
30.	*Timp3*	F- AGGATGCCTTCTGCAACTCCGA<br>R- GTGTAGACCAGAGTGCCAAAGG
31.	*Pai-1*	F - GAGGTGGAAAGAGCCAGATTTA<br>R- CCACTGAAGTAGAGGGCATTC
32.	*Mmp2*	F - CAAGGATGGACTCCTGGCACAT<br>R - TACTCGCCATCAColGCGTTCCCAT
33.	*Mmp7*	F - AGGTGTGGAGTGCCAGATGTTG<br>R - CCACTACGATCCGAGGTAAGTC
34.	*Mmp9*	F - GCTGACTACGATAAGGACGGCA<br>R - TAGTGGTGCAGGCAGAGTAGGA
35.	*Hai1*	F- CTTCGTGAGGAAGAGTGCATGC<br>R-TCACACTCCAGGAAGCCATCGA
36.	*Areg*	F CAGAAGAATGGAAGAGTCAG<br>R CAGATATGCAGGGAGTCACC
37.	*Opn*	F TGAGAGCAATGAGCATTCCGATG<br>R CAGGGAGTTTCCATGAAGCCAC
38.	*He4*	F - AGGTCAAGTCTCCACGAAGCCA<br>R - AGAACACTGGCTGTCCACCTGA
39.	*Ngal*	F - ATGTCACCTCCATCCTGGTCAG<br>R - GCCACTTGCACATTGTAGCTCTG
40.	*Kim1*	F-AAACCAGAGATTCCCACACG<br>R-GTCGTGGGTCTTCCTGTAGC
41.	*Fn1*	F -ATGTGGACCCCTCCTGATAGT<br>R -GCCCAGTGATTTCAGCAAAGG
42.	*Icam1*	F -CTTCCAGCTACCATCCCAAA<br>R -CTTCAGAGGCAGGAAACAGG
43.	*IL-6*	F- CTTCCATCCAGTTGCCTTCT<br>R- CTCCGACTTGTGAAGTGGTATAG
44.	*IL-10*	F-TTTGAATTCCCTGGGTGAGAA<br>R-ACAGGGGAGAAATCGATGACA
45.	*Tnf*	F-ACCCTCACACTCAGATCATCTTC<br>R-TGGTGGTTTGCTACGACGT
46.	*Ifn*	F -GGCCATCAGCAACAACATAAGCGT<br>R-TGGGTTGTTGACCTCAAACTTGGC
47.	*Ccl2*	F -CTCGGACTGTGATGCCTTAAT<br>R- TGGATCCACACCTTGCATTTA
48.	*Ccl3*	F-GAAGATTCCACGCCAATTCATC<br>R-GATCTGCCGGTTTCTCTTAGTC
49.	*IL1b*	F -TTGACGGACCCCAAAAGAT<br>R - GAAGCTGGATGCTCTCATCTG
50.	*Postn*	F-CAAAGCACACAGTTACCTTTCCAGGG<br>R-GCAGGAAACCCACATTGCATGAGA
