# Supplementary material for: Pirfenidone treatment attenuates fibrosis in autosomal dominant polycystic kidney disease
Source: JCI Insight. 2026 Sep 22;11(18):e207552. doi: 10.1172/jci.insight.207552 (PMC13596711; doi:10.1172/jci.insight.207552)

Figure 3H

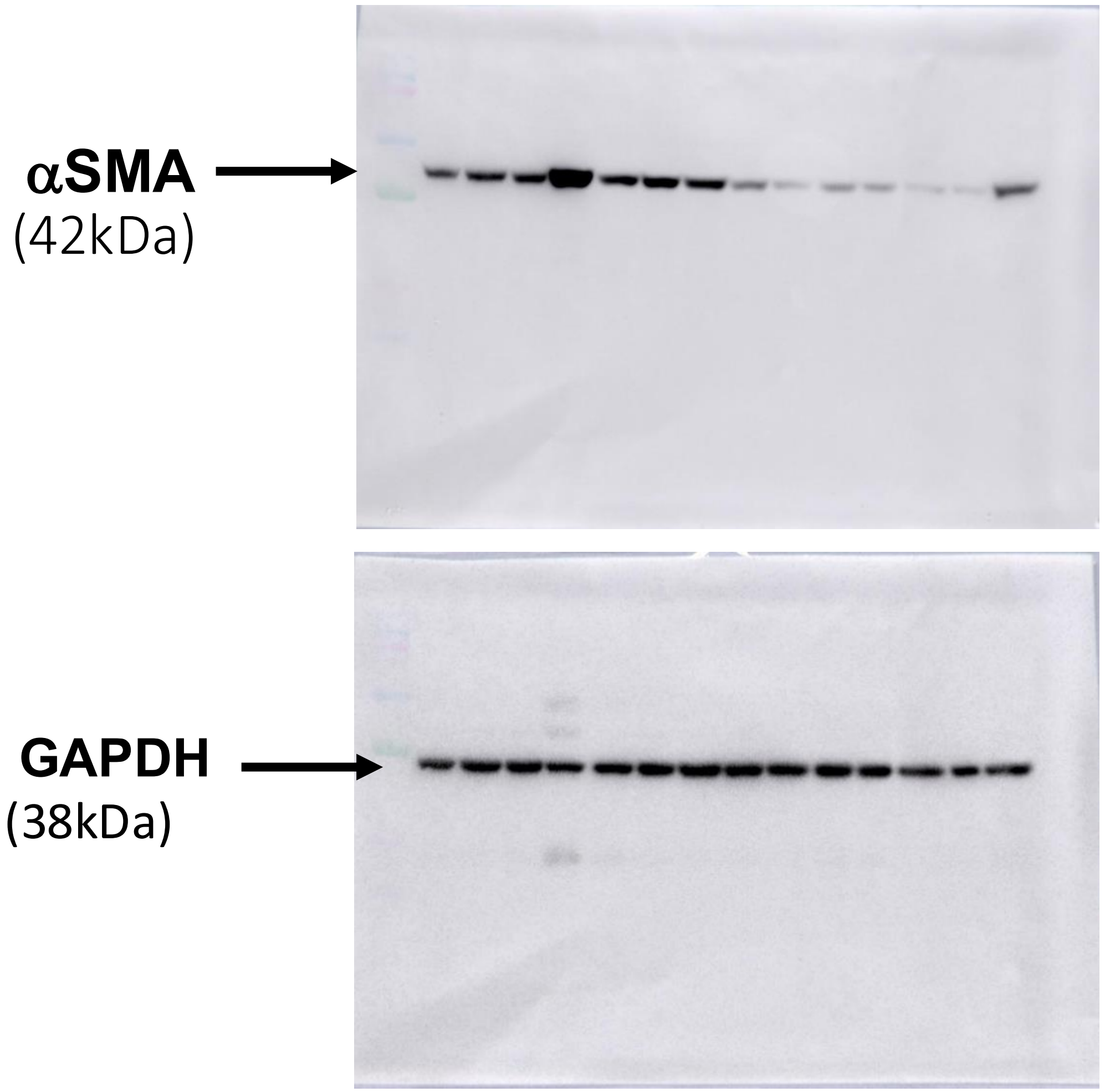

Figure 5A

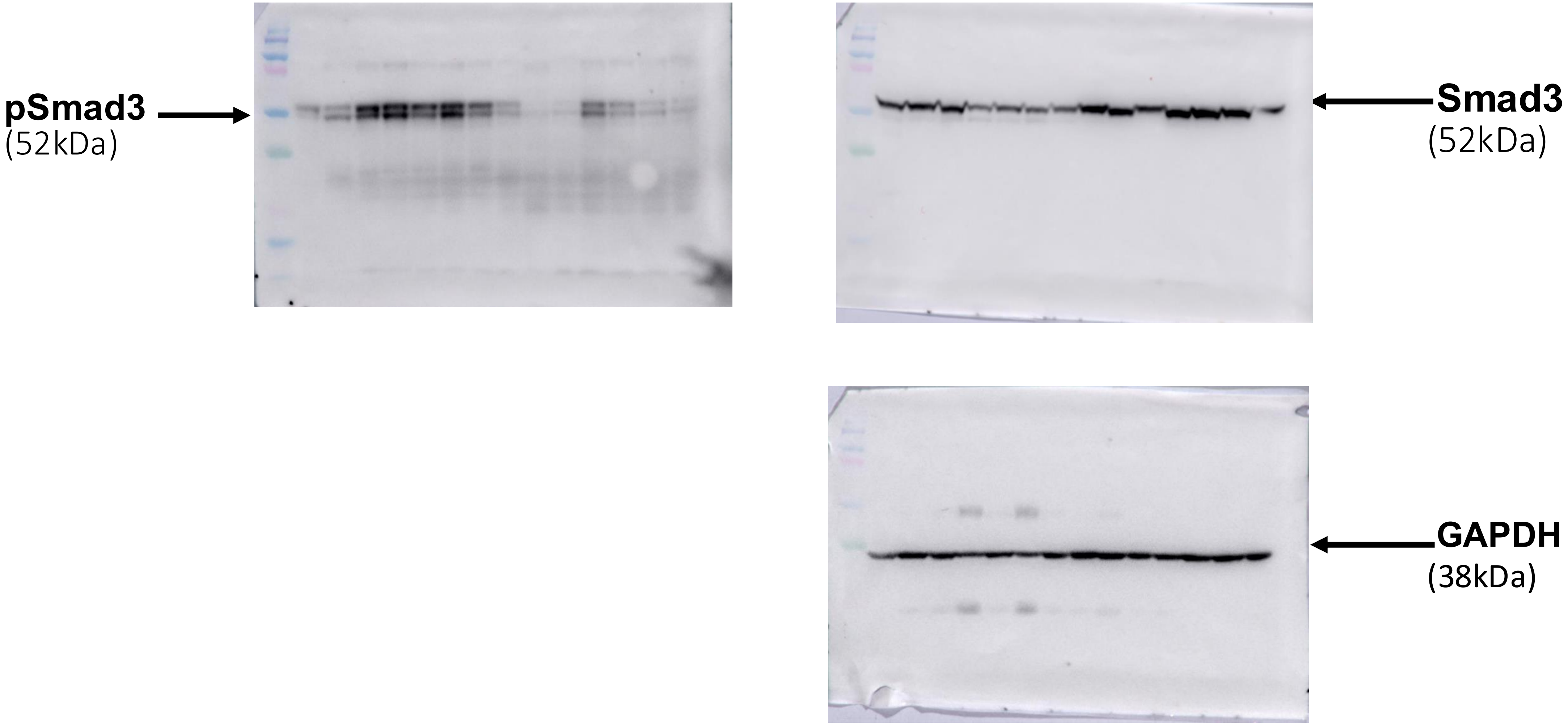

Figure 5A

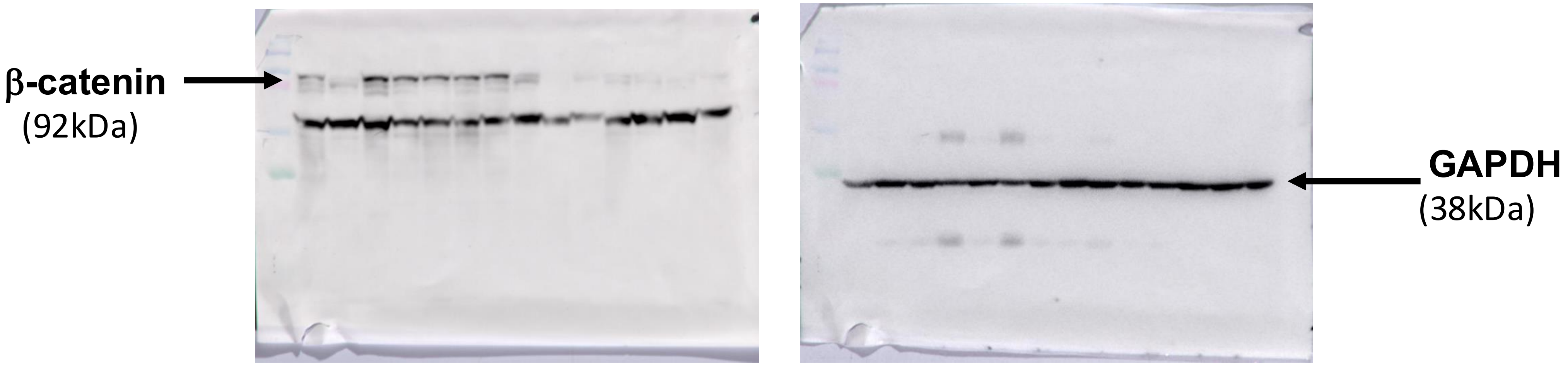

pSMAD, SMAD and βcatenin were probed on the same blot; therefore, the same GAPDH loading control is shown for both.

Figure 5C

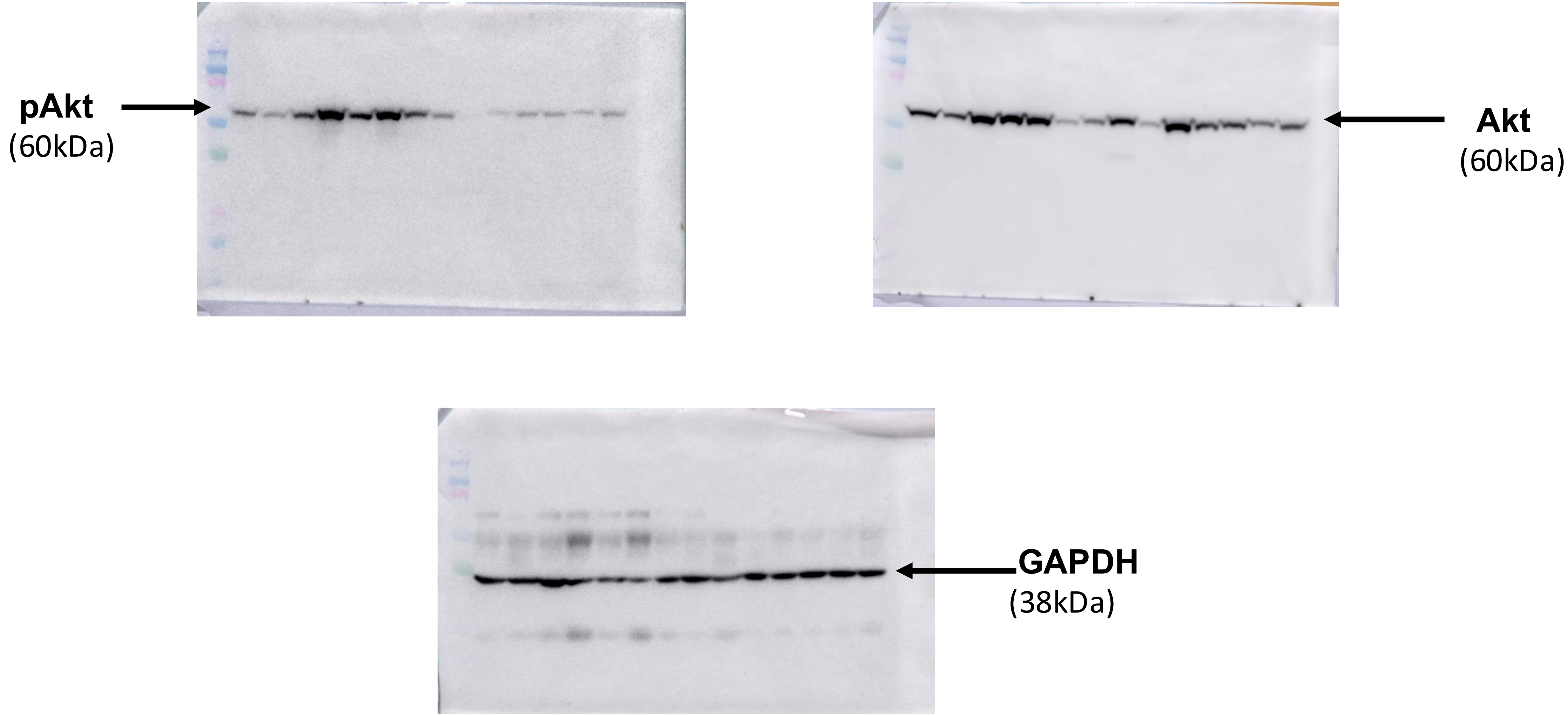

Figure 5C

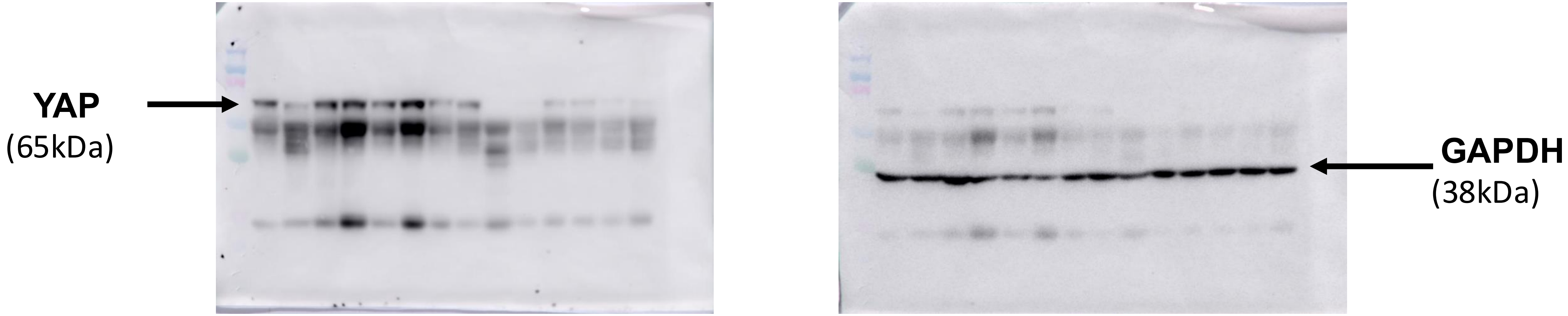

pAkt, Akt and YAP were probed on the same blot; therefore, the same GAPDH loading control is shown for both.

Figure 5C

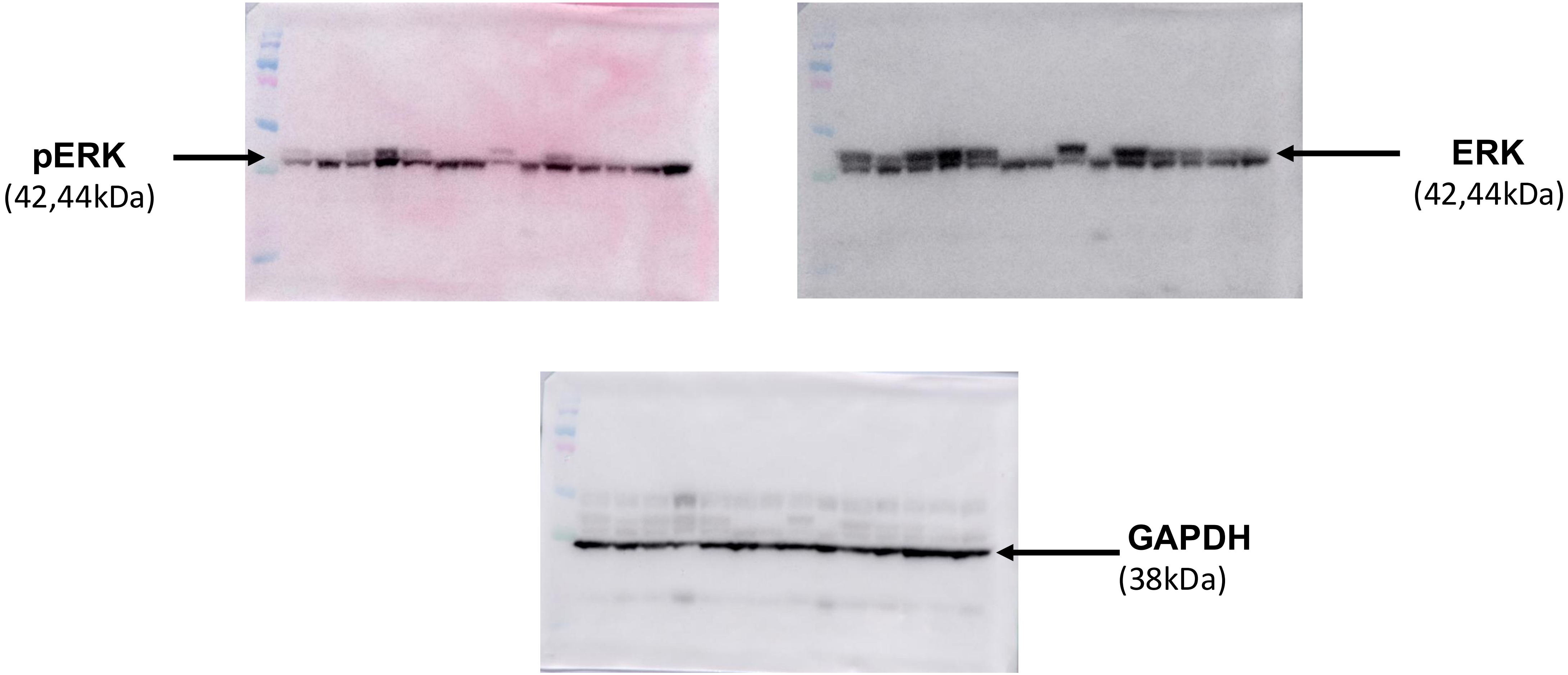

Figure 7C

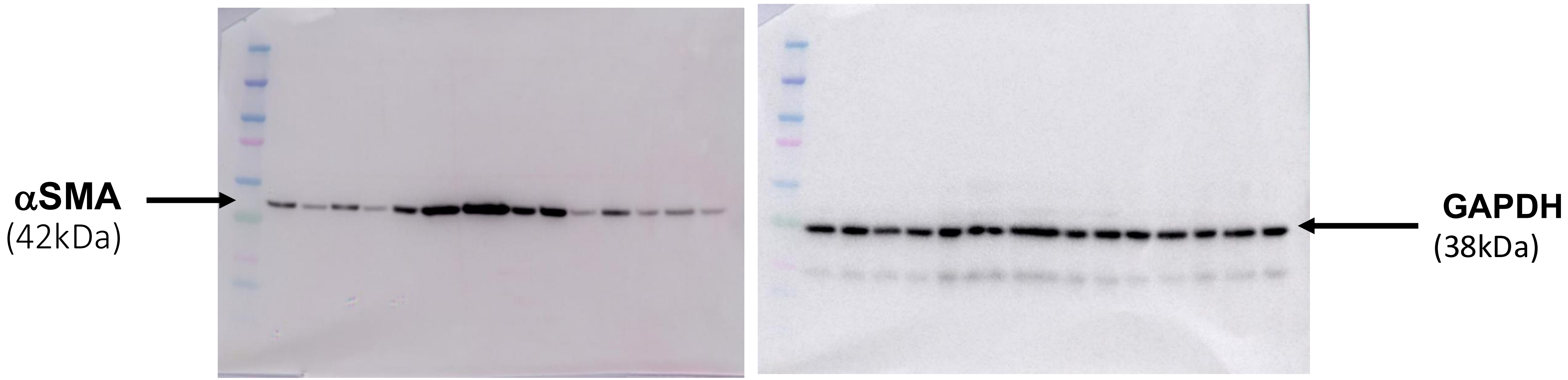

Figure 7F

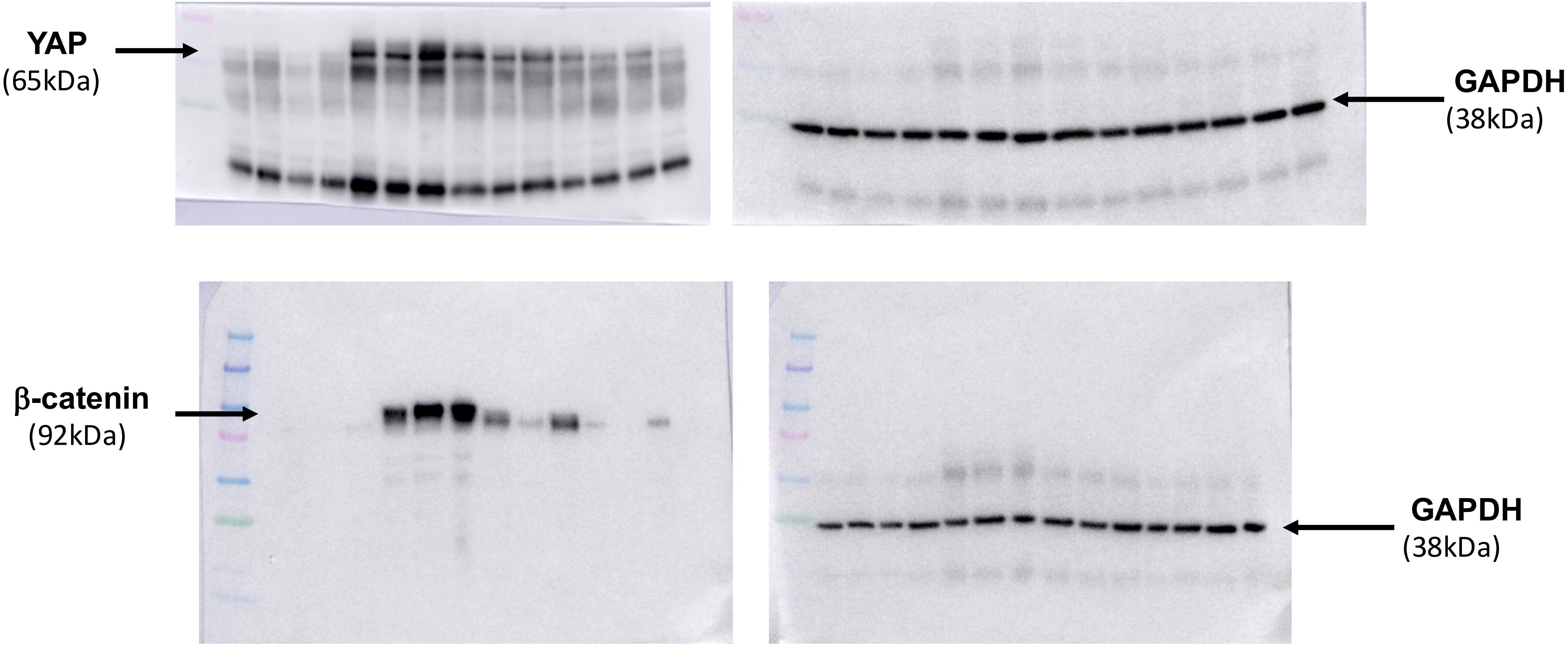

Figure 7F

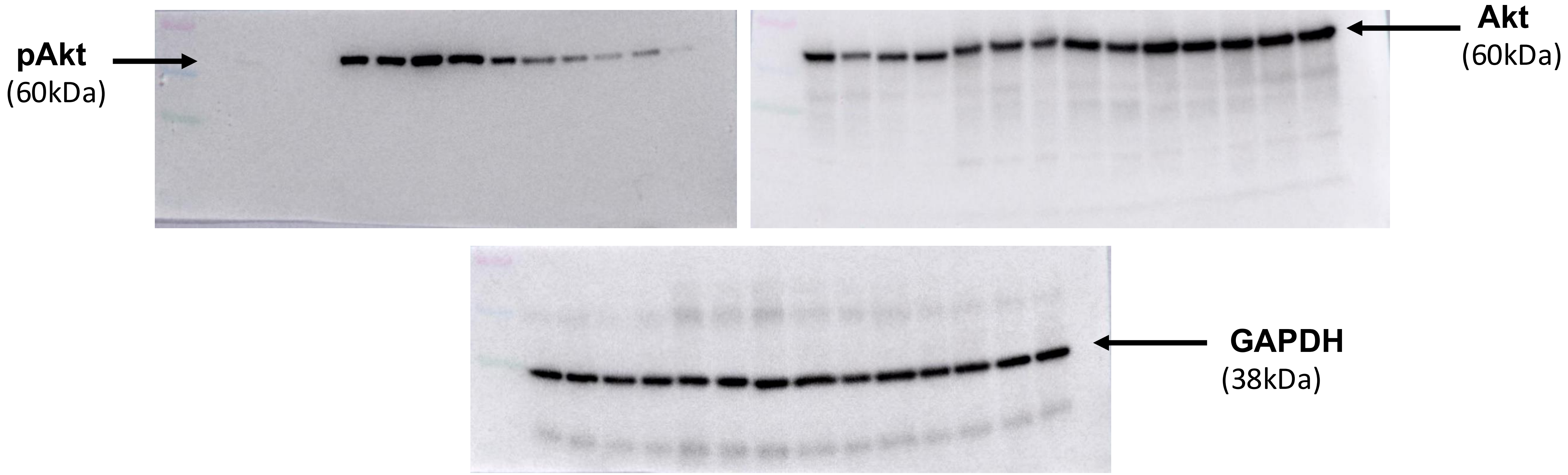

Figure 7F

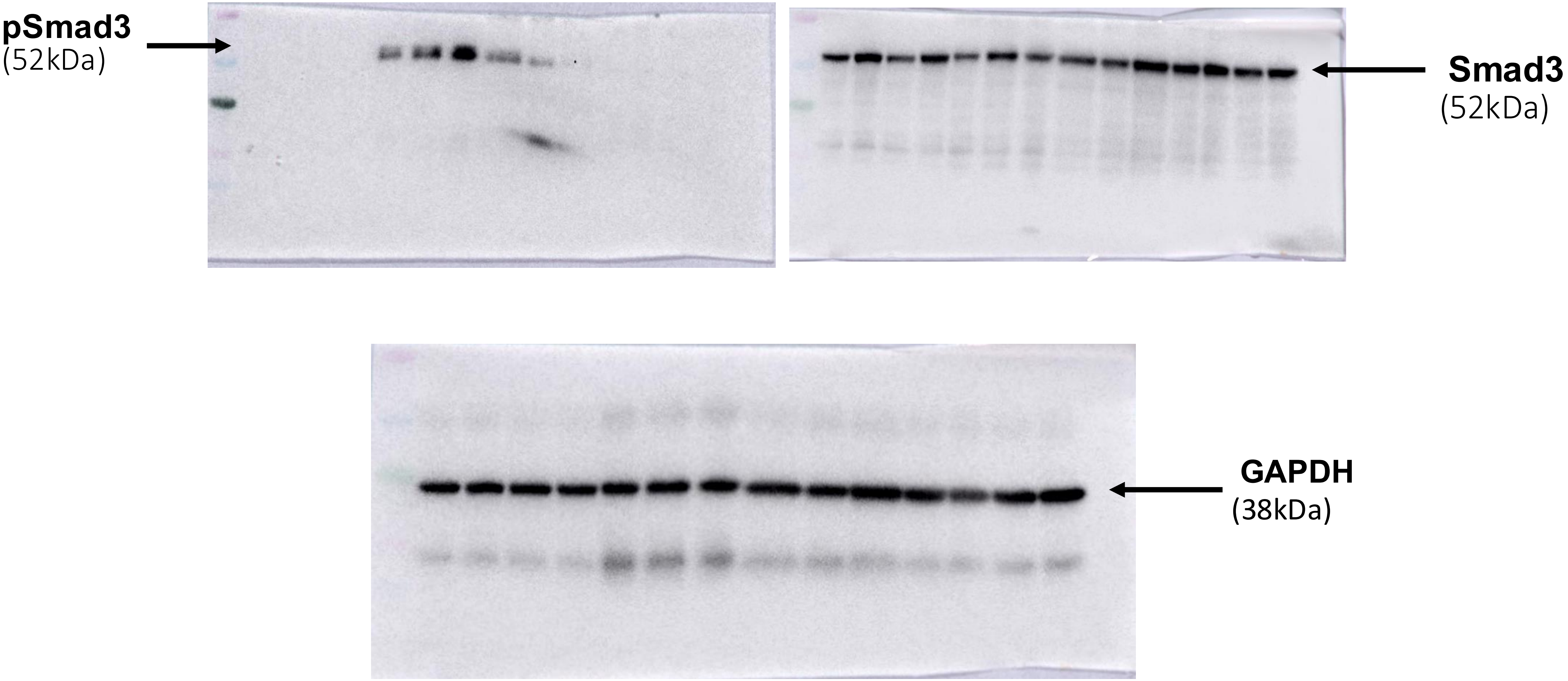

Supplemental Fig. 3

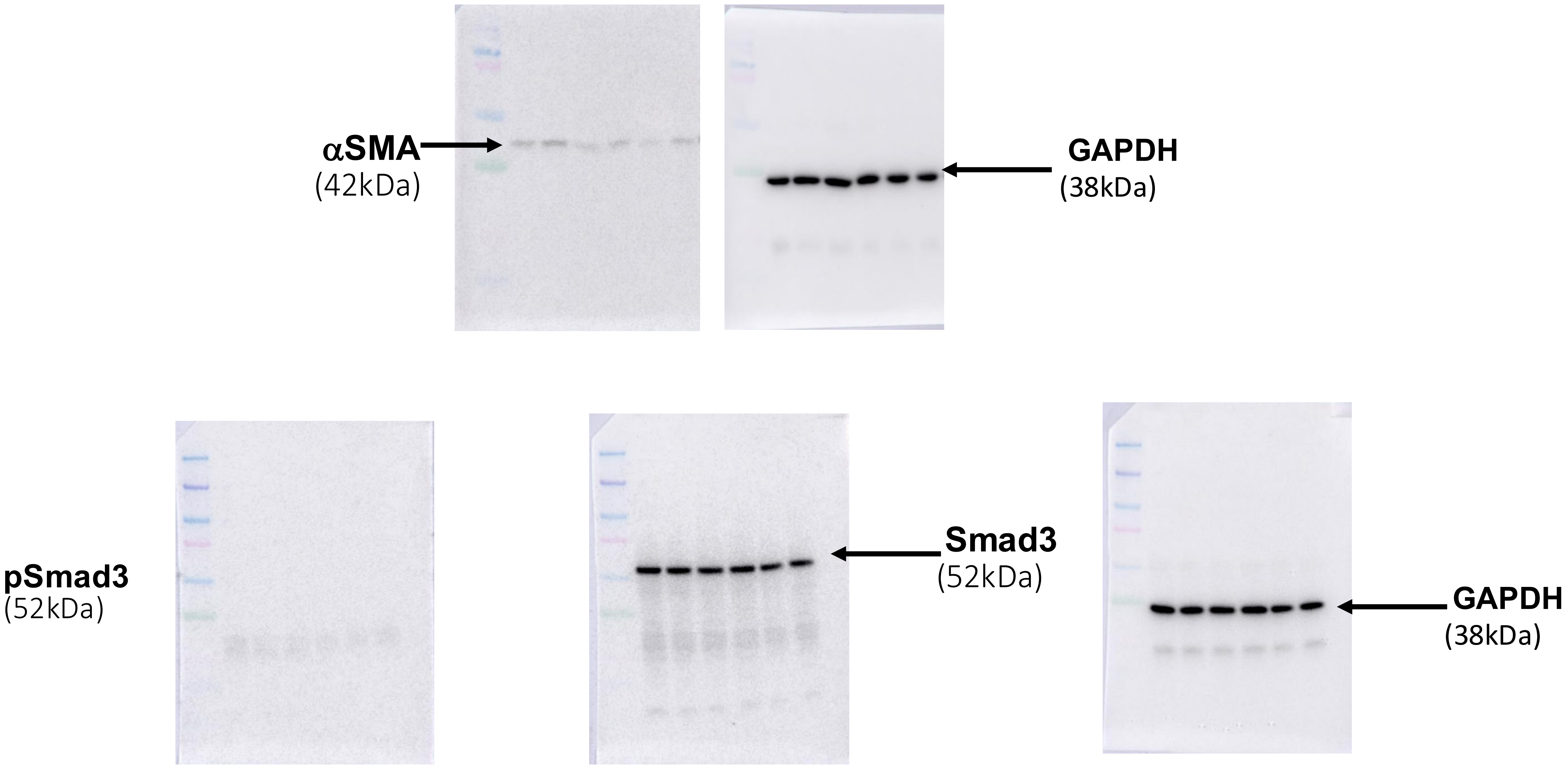

Supplemental Fig. 4

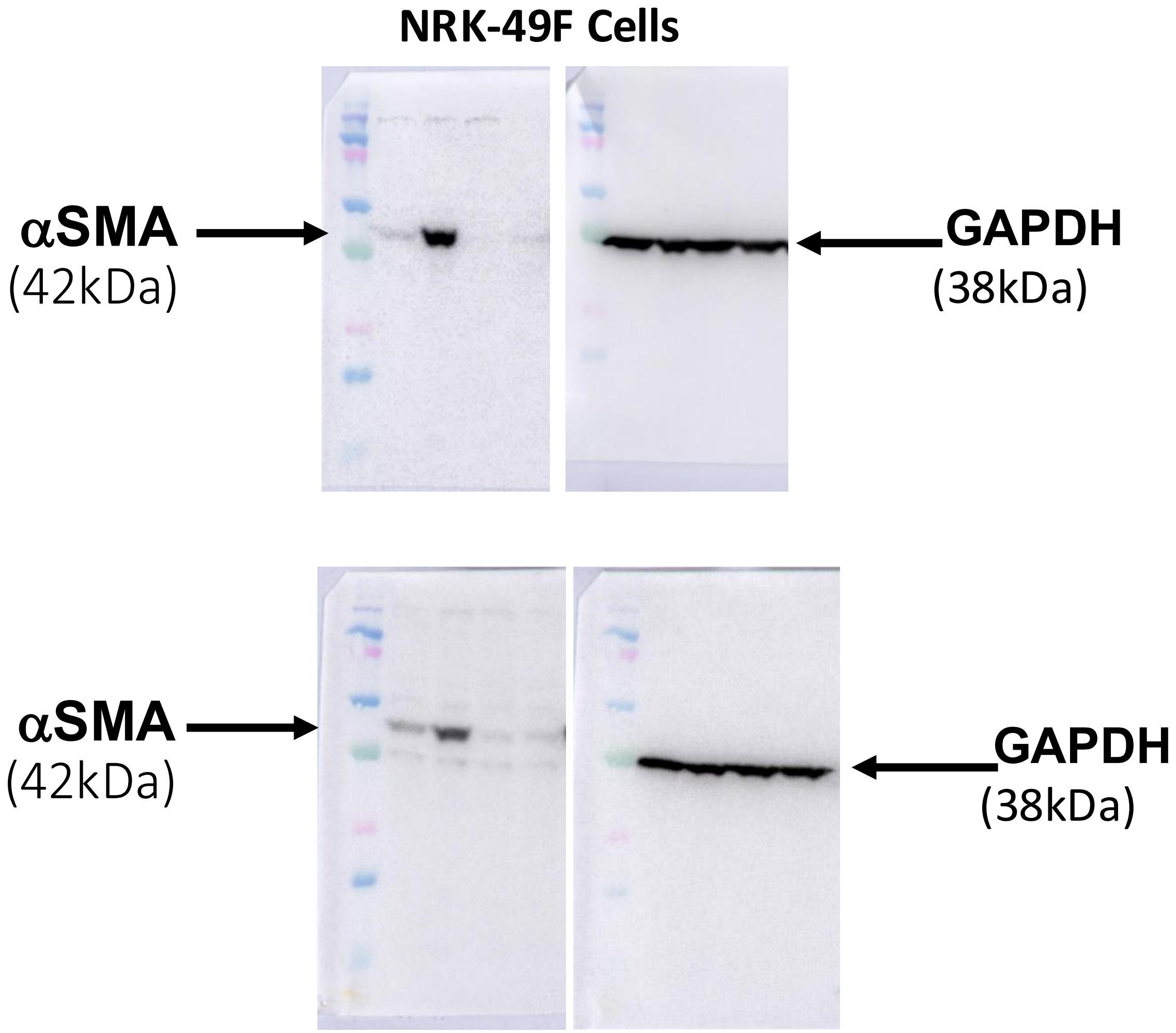

Supplemental Fig. 6

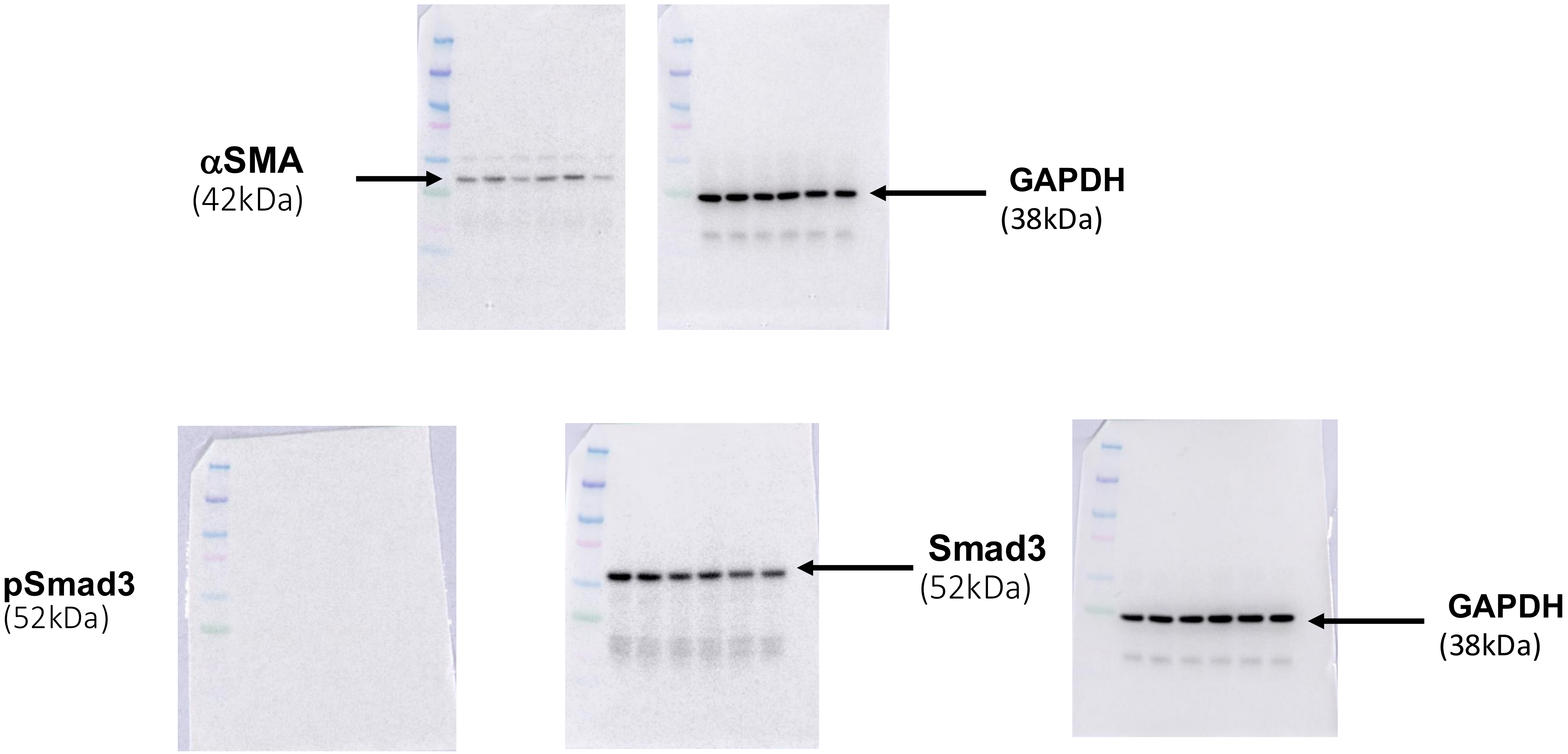

Supplement: Supplementary file 2 [file jciinsight-11-207552-s310.pdf]
